# Fragile futures: Evaluating habitat and climate change response of hog badgers (Mustelidae: *Arctonyx*) in the conservation landscape of mainland Asia

**DOI:** 10.1002/ece3.70160

**Published:** 2024-08-14

**Authors:** Imon Abedin, Tanoy Mukherjee, Ah Ran Kim, Soo Rin Lee, Hyun‐Woo Kim, Shantanu Kundu

**Affiliations:** ^1^ Agricultural and Ecological Research Unit Indian Statistical Institute Kolkata India; ^2^ Research Center for Marine Integrated Bionics Technology Pukyong National University Busan Republic of Korea; ^3^ Department of Marine Biology Pukyong National University Busan Republic of Korea; ^4^ Department of Biology, Faculty of Science and Technology Airlangga University Surabaya Indonesia; ^5^ Institute of Fisheries Science, College of Fisheries Sciences Pukyong National University Busan Republic of Korea; ^6^ International Graduate Program of Fisheries Science Pukyong National University Busan Republic of Korea

**Keywords:** conservation priorities, global warming, habitat suitability, mainland Asia, Mammalia, species distribution model

## Abstract

The small mammalian fauna plays pivotal roles in ecosystem dynamics and as crucial biodiversity indicators. However, recent research has raised concerns about the decline of mammalian species due to climate change. Consequently, significant attention is directed toward studying various big flagship mammalian species for conservation. However, small mammals such as the hog badgers (Mustelidae: *Arctonyx*) remain understudied regarding the impacts of climate change in Asia. The present study offers a comprehensive analysis of climate change effects on two mainland hog badger species, utilizing ensemble species distribution modeling. Findings reveal concerning outcomes, as only 52% of the IUCN extent is deemed suitable for the Great Hog Badger (*Arctonyx collaris*) and a mere 17% is ideal for the Northern Hog Badger (*Arctonyx albogularis*). Notably, projections suggest a potential reduction of over 26% in suitable areas for both species under future climate scenarios, with the most severe decline anticipated in the high‐emission scenario of SSP585. These declines translate into evident habitat fragmentation, particularly impacting *A. collaris*, whose patches shrink substantially, contrasting with the relatively stable patches of *A. albogularis*. However, despite their differences, niche overlap analysis reveals an intriguing increase in overlap between the two species, indicating potential ecological shifts. The study underscores the importance of integrating climate change and habitat fragmentation considerations into conservation strategies, urging a reassessment of the IUCN status of *A. albogularis*. The insights gained from this research are crucial for improving protection measures by ensuring adequate legal safeguards and maintaining ecological corridors between viable habitat patches, which are essential for the conservation of hog badgers across mainland Asia. Furthermore, emphasizing the urgency of proactive efforts, particularly in countries with suitable habitats can help safeguard these small mammalian species and their ecosystems from the detrimental impacts of climate change.

## Graphical abstract

The ensemble model unveils concerning outcomes for two mainland hog badgers, indicating a widespread decline in habitat across mainland Asia. The research underscores certain countries as focal points for conserving these small mammals and calls for reassessing both species in their extent.

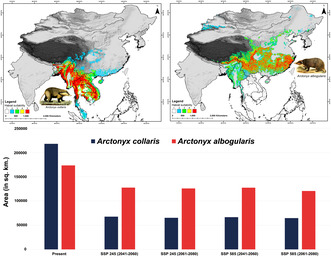

## 1 INTRODUCTION

In recent years, the global ecological balance has been significantly affected by climate change and a multitude of human‐induced pressures (Jha & Bawa, 2006; Kiene et al., 2021). The consequences of anthropogenic pressures, particularly alterations in land cover, have led to considerable deterioration and fragmentation of habitats within natural ecosystems inhabited by a diverse range of mammalian fauna (Ripple et al., 2015). Such impacts have driven nearly half a million species to the verge of extermination, resulting in the ongoing sixth mass extinction worldwide (Ceballos et al., 2015; Conde et al., 2019; Mokany et al., 2020). Hence, considerable focus is placed on the study of various aspects of mammalian biology, with particular emphasis on the preservation of remaining natural habitats to mitigate further extinctions (Rondinini et al., 2011). However, this attention tends to be predominantly directed toward flagship mammalian species families (Pant et al., 2021; Rather et al., 2020; Xu et al., 2024). Thus, smaller carnivores, lacking in such charismatic appeal, receive disproportionately less attention in terms of research and conservation efforts, despite their importance for safeguarding keystone habitats (Kalle et al., 2013). Additionally, these small mammals exhibit high sensitivity to landscape characteristics, making them valuable indicators of the relationship between land use and biodiversity (Paniccia et al., 2022). These small mammals play crucial roles in ecosystem processes and contribute to key ecological mechanisms such as seed and spore dispersal, pollination, soil nutrient cycling, and energy flux regulation (Hurst et al., 2014). Moreover, the high abundance and diversity of small mammals are considered reliable indicators of sustainable forest management. Consequently, they serve as excellent models for studying evolutionary processes within ecosystems under changing environmental conditions and habitat vulnerabilities (Chirichella et al., 2022).

One such mammalian genera deserving of research attention is *Arctonyx* (Mammalia: Mustelidae), for which current knowledge regarding its ecology and distribution range is notably limited (Helgen et al., 2008; Proulx et al., 2016). The genera *Arctonyx* encompasses badgers, primarily represented by three species: Great Hog Badger (*Arctonyx collaris*), Northern hog Badger (*A. albogularis*), and the Sumatran Hog Badger (*A. hoevenii*; Cao et al., 2023). They represent a group of small to medium‐sized carnivores found across East and Southeast Asia, spanning regions including much of China, the eastern Indian Subcontinent, Indochina, and the lone Indonesian island of Sumatra. The genus *Arctonyx* was previously considered monotypic with consists of a single species *A. collaris*. Later, based on a comprehensive examination of available museum collections this genus was further segregated into three distinct species (Helgen et al., 2008; Proulx et al., 2016). This recognition is based on craniometric analyses, qualitative craniodental characteristics, external morphological comparisons, as well as geographical and ecological factors.

The Northern hog Badger, *A. albogularis* is characterized by a shaggy coat and is of medium size, exhibiting a wide distribution across temperate Asia, from Tibet and the Himalayan region to eastern and southern China. On the other hand, the Great hog badger, *A. collaris*, is notably large with shorter hair and is found throughout Southeast Asia, ranging from eastern India to Myanmar, Thailand, Vietnam, Cambodia, and Laos (Duckworth et al., 2016). Interestingly, the species was mapped as Possibly Extinct in Vietnam in the IUCN Red List of Threatened Species, though the text account states that the species was considered extant in the country at the time of its assessment in 2015 (Cao et al., 2023). Moreover, the Sumatran hog badger, *A. hoevenii*, represents the smallest and darkest member of the genus, being endemic to the Barisan mountain range of Sumatra, Indonesia. Other than *A. hoevenii*, no other species of *Arctonyx* are found on the Island ecosystem (Holden et al., 2016). More interestingly, the largest existing badger in the world, *A. collaris* shares the overlapping distribution with *A. albogularis* in Mainland Asia (northeastern India and uncertain in southern China).

Both the mainland hog badgers have a versatile habitat across their distribution range in South and Southeast Asia. The *A. collaris* demonstrates a broad habitat range, extending from dense forests (both deciduous and evergreen) to open landscapes, including grassland‐dominated floodplains in northeastern India within the elevation range up to 2300 m above sea level (Choudhury, 2013). In Southeast Asia, sightings of *A. collaris* primarily occur within forested areas, although the extent of their presence in nonforest habitats remains uncertain (Duckworth et al., 2016). Despite limited knowledge regarding their dietary habits, there is speculation that they may possess a predominantly vermivorous diet, inferred from morphological characteristics rather than direct dietary observations (Helgen et al., 2008). On the other hand, Northern Hog Badger exhibits a remarkable elevational distribution, ranging from sea level to at least 4300 m in China. It has been observed in both forested regions and grasslands, displaying an omnivorous diet that includes invertebrates, small vertebrates, and plant material (Zheng et al., 1988). These mammals are primarily nocturnal, seeking refuge in burrows they excavate themselves or in natural shelters (Duckworth et al., 2016; Pocock, 1941). However, both species face significant threats from habitat alteration and fragmentation, which are prevalent in Southeast Asia. Additionally, hunting poses a substantial impact on the species throughout its range, with their hairs sought after for European shaving brush production (Domingo‐Roura et al., 2006). Hence, numerous studies have drawn attention to the inadequate knowledge of their range and habitat requirements, recommending focused research initiatives to guide conservation plans for mainland hog badgers throughout their range (Cao et al., 2023; Helgen et al., 2008; Proulx et al., 2016).

In recent years, there has been rapid progress and widespread use of species distribution modeling (SDMs) for evaluating habitat suitability (Loiseau et al., 2020; Mohammadi et al., 2024; Ye et al., 2018). This method enables the mapping of species distribution patterns and the quantitative evaluation of the impact of various environmental factors (Elith & Leathwick, 2009). This approach has effectively delineated habitat suitability for numerous similar species under the family Mustelidae worldwide, as evidenced by several studies (Almasieh & Cheraghi, 2022; Barlow et al., 2021; Schiaffini et al., 2013). Moreover, the ongoing environmental crisis in Southeast Asia not only impacts the distribution of this small mammalian group but also impairs the fragmented populations' ability to respond to further environmental alterations induced by climate change (Radchuk et al., 2019). Consequently, numerous experts have identified both direct and indirect effects of climate change on small mammalian species, highlighting this method of habitat modeling and climate change response as high priorities for future research (Droghini et al., 2022). Therefore, the ensemble model was manifested to be a powerful method for estimating the habitat suitability of various species (Hao et al., 2020). This ensemble approach uses multiple modeling algorithms to predict the distribution of species across geographic areas. The rationale behind this integrated approach involves combining different approaches used by different models that may capture diverging aspects and underlying processes governing the distribution. Hence, this approach intends to balance the strengths and weaknesses of individual models, resulting in improved accuracy and reliability of predictions. This approach was further demonstrated to assess the impacts of climate change and habitat fragmentation, as well as to analyze the niche breadth and overlap between the sister species (Dutta et al., 2022). Thus, forecasting species responses to climate change has become crucial across multiple disciplines in biodiversity research, making the implementation of SDM with ensemble approach increasingly important for assessing climate sensitivity and the potential impacts of climate change (Morueta‐Holme et al., 2010; Rowe & Terry, 2014). Although, the conservation status of *A. collaris* is assessed by the IUCN/SSC small mammal specialist group (SMSG) and categorized as “Vulnerable,” however, the knowledge of its suitable extent in South and Southeast Asia is still lacking to the scientific communities for precise conservation planning in response to current and future environmental crisis. Further, another mainland species, *A. albogularis*, and island species *A. hoevenii* are categorized as “Least Concern” in the IUCN Red List. Given that both mainland badger species (*A. collaris* and *A. albogularis*), exhibit overlapping ranges while the island species (*A. hoevenii*), inhabits a confined range, this study exclusively focused its ecological evaluation on the two mainland hog badger species.

Therefore, this study aimed to employ an ensemble approach utilizing specific variables based on IUCN criteria for both mainland badger species across all mainland countries (Bangladesh, Bhutan, Cambodia, China, Laos, India, Myanmar, Thailand, and Vietnam) and adjoining mainland landmasses (Malaysia and Korean Peninsula) comprising similar habitat within their known IUCN ranges to: (i) ascertain the current distribution and the impacts of climate change on their habitats; (ii) evaluate habitat fragmentation under future climatic scenarios; and (iii) assess the niche breadth and niche overlap. The findings of the current study will assist in identifying specific priority sites as well as informing conservation decisions regarding the hog badgers and related mustelid species. Thus, this study will address the inadequate knowledge regarding their habitat requirements and the extent of suitable habitats of the mainland badgers. This will facilitate the initiation of effective conservation measures and ensure the long‐term monitoring of these small mammalian species in the context of present and future climate change scenarios in South and Southeast Asia.

## 2 MATERIALS AND METHODS

### 2.1 Study area and species occurrence records

**FIGURE 1:**
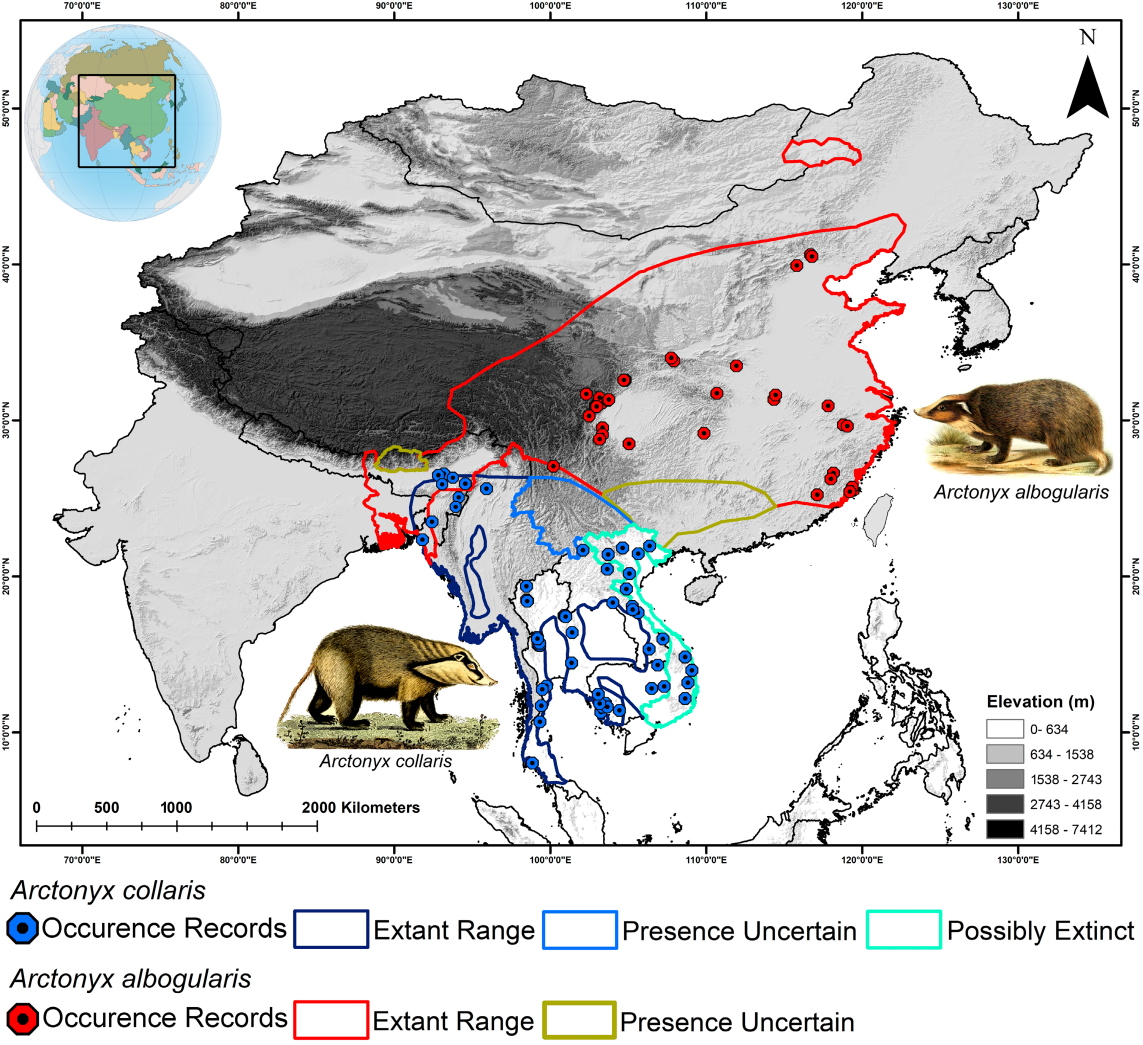
Map showing the study area for the present study along with the IUCN extant of Great Hog Badger (*Arctonyx collaris*) and Northern Hog Badger (*A. albogularis*). The figure also highlights the location points collected for the modeling. The artworks of the mainland hog badgers are reproduced from the free online media repositories FreeSVG.org and Wikimedia Commons.

The IUCN range of *A. collaris* extends from Bangladesh and north‐east India eastward through Myanmar, Thailand, and Laos to Vietnam, and southward to Cambodia and peninsula Thailand; it likely also includes Yunnan province, China, encompassing lowland forested areas across Southeast Asia (Helgen et al., 2008). In contrast, *A. albogularis* inhabits higher elevations in north‐east India and presumably Bhutan extending across southern and eastern China, from Gansu, Hebei, Shanxi, and Liaoning provinces in the north to Yunnan, Guangxi, and Guangdong in the south. The study area was selected to encompass all the mainland countries where the IUCN identifies the two mainland hog badger species and adjoining mainland landmasses (Malaysia and Korean Peninsula) comprising similar habitats in South and Southeast Asia. This approach ensures a comprehensive delineation of suitable habitat extent for both species across each mainland country within the IUCN's defined extent. This approach facilitates the prediction of suitable habitats capable of accommodating both species. In the last 5 years (2019–2023), opportunistic field surveys have been conducted in the northeastern region of India, specifically focusing on two states, Assam and Manipur. Despite their elusive nature, *A. collaris* was sighted in Kaziranga National Park, Assam (location points, *n* = 11) and Bunning Wildlife Sanctuary, Manipur (location points, *n* = 2). The location points were collected using the Garmin GPS eTrex 10 and photographic records were taken using Canon 7D MK 2 with Canon 100–400 mm Lens (Figure S1). Additionally, the study utilized location points from the available literatures of *A. collaris* (*n* = 16) (Cao et al., 2023; Helgen et al., 2008; Kundu et al., 2019), secondary citizen science platforms GBIF (2024a, 2024b; *A. collaris* = 81, https://doi.org/10.15468/dl.uh64a9; *A. albogularis* = 35, https://doi.org/10.15468/dl.wat8ku) and iNaturalist (*A. collaris* = 45; *A. albogularis* = 20; Figure 1). However, during the retrieval of data from the citizen science website, it was ensured that records of museum specimens or individuals in captivity were excluded from the dataset. Additionally, the points outside of the IUCN extent have been excluded from the final dataset. This was done to enhance the reliability of the dataset and accurately represent the ecological areas of interest. The final models were developed using only 108 locations for *A. collaris* and 55 locations for *A. albogularis*, following the removal of spatial correlation between the occurrences using the spatial rarefy occurrence point function in SDM Toolbox v2.4 (Brown et al., 2017). The spatial correlation between occurrences was conducted at a resolution of 4.5 km^2^. This specific resolution was chosen to align with the size of one pixel in the raster data, thereby reducing the risk of overfitting the model and ensuring a more accurate analysis (Abedin, Mukherjee, Kang, et al., 2024).

### 2.2 Selection of covariates for the habitat suitability assessment

A comprehensive set of covariates, which included bioclimatic, topographic, habitat, and anthropogenic variables, was chosen to analyze their effect on both species. The bioclimatic variables (*n* = 19) were acquired from the WorldClim website (https://www.worldclim.org/) (Su et al., 2018). Meanwhile, the topographic variables (elevation, slope, and aspect) were obtained from the Diva‐GIS website (http://srtm.csi.cgiar.org/srtmdata/) at a spatial resolution of 90 meters. To assess the effect of different habitat types on both species, variables are chosen as per criteria outlined by the IUCN Red List (Duckworth et al., 2016; Helgen & Chan, 2016). Therefore, habitat variables such as the normalized difference vegetation index (NDVI), evergreen needle leaf closed forest (euc_111), evergreen broad leaf closed forest (euc_112), deciduous broad leaf closed forest (euc_114), shrubs (euc_20), and herbaceous wetlands (euc_90) were chosen. Additionally, the anthropogenic variable built‐up/urban (euc_built) was included to evaluate its impact on the species extent. Furthermore, all land use and land cover (LULC) class variables derived from the Copernicus Global Land Service were converted into continuous raster datasets using the Euclidean distance function in ArcGIS, to assess the importance of each class and how the species reacts to its proximity (Buchhorn et al., 2020). It is crucial to note that various species exhibit a strong preference for residing in proximity to forest types that offer suitable conditions for their survival and well‐being (Mukherjee, Sharma, et al., 2021). Subsequently, all variables were resampled to 2.5 min (~4.5 km^2^) using the spatial‐analyst extension in ArcGIS 10.6. The spatial multicollinearity testing among the variables was performed using the SAHM (Software for Assisted Habitat Modeling) package in the VisTrails software (Morisette et al., 2013). Covariates exhibiting a correlation (*r*) greater than .8 were excluded from the analysis (Warren et al., 2010; Figures S2 and S3). Therefore, after addressing the correlation among the variables, a total of 14 variables were retained for the final model for both species.

### 2.3 SDM development for the mainland hog badgers

The assessment of distribution models involved the utilization of multiple modeling algorithms through an ensemble approach to formulate the final distribution model for both species. Hence, five distinct algorithms—maximum entropy (MaxEnt), random forest (RF), boosted regression tree (BRT), generalized linear model (GLM), and multivariate adaptive regression splines (MARS)—were employed (Elith & Leathwick, 2009; Guisan et al., 2007; Miller, 2010). These algorithms were executed in the VisTrails software using the SAHM package (Morisette et al., 2013; Talbert & Talbert, 2012). The execution produced probability surfaces ranging from 0 (indicating least suitability) to 1 (indicating highest suitability), and binary maps were generated using the minimum training presence as the threshold. The model evaluation relied on an area under the curve (AUC) threshold of 0.75 as the elimination criteria for the selected models (Hayes et al., 2015; Lavazza et al., 2023; Salas et al., 2018). The ensemble count map was constructed on a scale from 0 to 5, where each pixel denoted the number of model agreements, with a value of 5 indicating unanimous agreement across all five models, facilitating habitat configuration analysis. Additionally, to assess and compare model performance, various evaluation metrics, including area under the curve (AUC), true skill statistic (TSS), Cohen's Kappa, proportion correctly classified (PCC), specificity, and sensitivity, were calculated for both the training data and cross‐validation sets (*n* = 10; Allouche et al., 2006; Cohen, 1968; Jiménez‐Valverde et al., 2013; Phillips & Elith, 2010).

Furthermore, to project potential climate change scenarios across two distinct shared socioeconomic pathways (SSP)—namely ssp245 and ssp585—spanning the periods 2041–2060 and 2061–2080, future projections were used. The SSPs are scenarios used in climate change research to explore future socioeconomic conditions and their implications for greenhouse gas emissions and climate impacts. The SSP245 represents a future where moderate efforts are made to mitigate emissions and adapt to climate change and assumes moderate population growth, technological development, and a balanced approach to environmental and social policies. Here, greenhouse gas emissions increase gradually over the 21st century, stabilizing toward the end of the century with international cooperation on climate policies, albeit with challenges in implementation (O'Neill et al., 2014). Conversely, SSP585 depicts a future with high greenhouse gas emissions and limited adaptation efforts and assumes rapid population growth, high energy demand, and minimal environmental regulation, leading to continued emission increases throughout the century. This scenario reflects a world where there is little international cooperation on climate policies and insufficient societal efforts to mitigate emissions (Riahi et al., 2017). Furthermore, the study utilized the Hadley Centre Global Environment Model in Global Coupled Configuration 3.1 (HadGEM3‐GC31 LL), the sixth Coupled Model Intercomparison Project (CMIP6) (Andrews et al., 2020). The selection of this general circulation model (GCM) was based on its recognized performance in South and Southeast Asia and its ability to capture temporal fluctuations and excel in representing temperature distribution, as evidenced by previous research (Desmet & Ngo‐Duc, 2022; Norgate et al., 2024). For the present study, nonclimatic raster data, including various habitat types (such as evergreen needle leaf closed forest, evergreen broad leaf closed forest, deciduous broad leaf closed forest, shrubs, and herbaceous wetlands), NDVI, and built‐up/urban areas, remained constant in the future projections (Atsawawaranunt et al., 2024). This deliberate decision aimed to isolate the impact of climate change on the objective of the study. The approach constrained distribution probabilities within potential habitat zones in the study area and excluded projected regions like high ice‐capped mountains and barren plateau areas from consideration (Abedin, Mukherjee, Kim, et al., 2024). Furthermore, to facilitate the development of an effective conservation action plan, a comprehensive assessment of habitat suitability was conducted on a national scale, as there is a distinct legal framework for each country. This qualitative assessment of suitable habitats across different countries in their distribution range was conducted using the zonal statistics function in ArcGIS v.10.6 (Mukherjee, Sharma, et al., 2021).

### 2.4 Assessment of habitat quality and niche for the mainland hog badgers

To assess the qualitative and geometric characteristics of suitable patches in both current and projected future scenarios, various class‐level metrics were employed utilizing FRAGSTATS version 4.2.1 (McGarigal & Marks, 1995). It is a specialized software for landscape ecology, urban planning, and environmental management for analyzing spatial patterns in landscapes and ecosystems, providing a suite of metrics and indices to quantify and understand landscape structure and composition. The metrics encompassed in this study were the number of patches (NP), aggregate index (AI), patch density (PD), largest patch index (LPI), edge density (ED), total edge (TE), and landscape shape index (LSI). These indices such as NP, PD, ED, TE, and LPI provide detailed information about the geometry of the patches, including their size, edge characteristics, and density within a region. In contrast, LSI focuses on the shape complexity of the patches, indicating how convoluted or irregular the patches are, whereas AI measures the degree of proximity or clustering among patches, reflecting how aggregated or dispersed the patches are within the landscape. These metrics hold biological significance, shedding light on habitat ecological processes and offering valuable insights into the impacts of changes in suitable areas on landscape dynamics (Barwicka et al., 2021; Midha & Mathur, 2010). This methodology facilitates a deeper understanding of landscape characteristics and enables comprehensive analysis across the distribution range of the species. Consequently, these metrics were utilized to evaluate habitat features and levels of fragmentation in the modeled area across various scenarios, including present conditions and future climate change projections (Abedin, Mukherjee, Kang, et al., 2024; Kundu et al., 2023).

Likewise, the niche breadth and niche overlap of both mainland hog badger species were computed using the ENM tool Ver.1.3 (Warren et al., 2010). The niche breadth metrics assess the evenness of suitability scores across the geographic distribution predicted by a model. This approach evaluates model accuracy by employing Latin hypercube sampling to cover all environmental conditions within the range defined by the minimum and maximum values for each predictor variable in the training area. Similarly, Schoener's *D* was computed to assess the degree of niche overlap between the two hog badger species using this tool. This ecological niche modeling tool is specifically designed to conduct the niche overlap test and explore any potential niche radiation between species. The niche similarity test, based on Schoener's *D* evaluates the estimated habitat suitability results derived from a multi‐model ensemble. Schoener's *D* evaluates the suitable range for the studied species based on probability distributions in a georeferenced space (cells). A value of “0” indicates no overlap between species in terms of environmental factors, whereas a value of “1” signifies equal suitability of geographic space cells for both species (Mukherjee, Chongder, et al., 2021; Schoener & Gorman, 1968).

## 3 RESULTS

### 3.1 Distribution model performance

**FIGURE 2:**
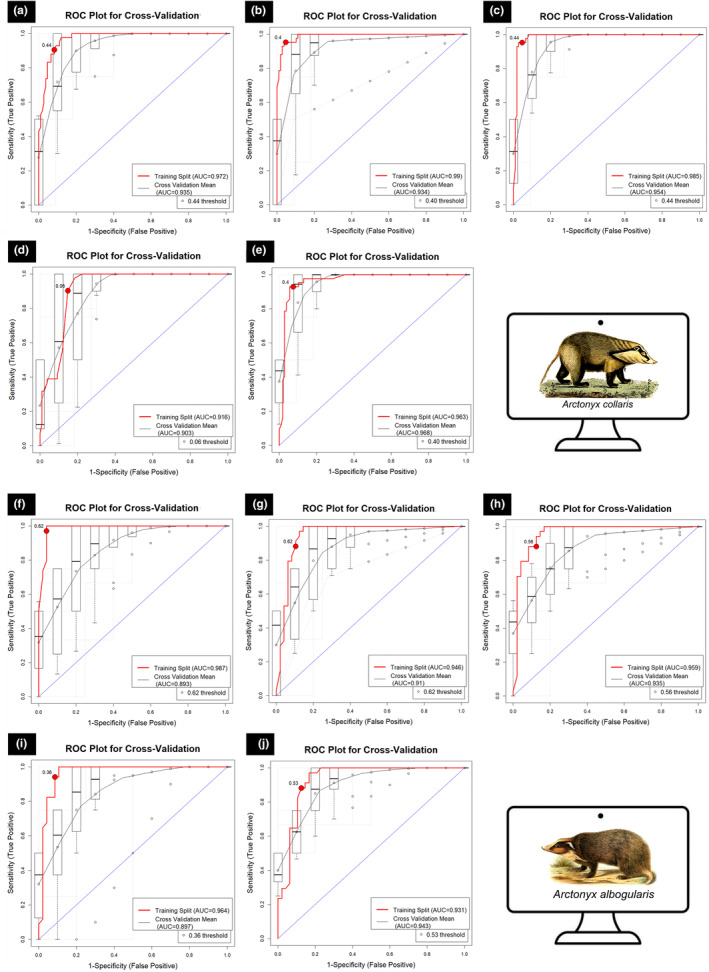
Model evaluation plot, showing the average training ROC of both training and cross‐validation (CV) for the replicate runs under five models of *Arctonyx collaris* and *A. albogularis*. For, *A. collaris*: (a) showing ROC plot of boosted regression tree (BRT), (b) generalized linear model (GLM), (c) multivariate adaptive regression splines (MARS), (d) maximum entropy (MaxEnt), and (e). Random forest (RF). For, *A. albogularis*: (f) showing ROC plot of boosted regression tree (BRT), (g) generalized linear model (GLM), (h) multivariate adaptive regression splines (MARS), (i) maximum entropy (MaxEnt), and (j) random forest (RF).

The resulting model performance for both species across all participating models exhibited excellence on both training and cross‐validation datasets. Since the model threshold of AUC (>0.75) was maintained for the final selection of the algorithms, all models were included in the final ensemble map. The models demonstrated an AUC range of 0.916–0.992 in training and between 0.903 and 0.968 in cross‐validation for *A. collaris*, while the AUC range for *A. albogularis* was 0.931–0.987 in training and between 0.893 and 0.943 in cross‐validation (Table 1, Figures 2 and S4–S7). The ΔAUC value exhibited the smallest difference for RF, with a value of 0.005 recorded for *A. collaris*, whereas GLM showed the highest ΔAUC values, reaching 0.056 across the replicate runs. Similarly, the ΔAUC value was smallest for RF once again, registering a value of 0.012 for *A. albogularis*, while the highest ΔAUC values were observed for BRT, reaching 0.094 across the replicate runs. These findings collectively highlight the sensitivity of the data employed for model fitting across all models. The evaluation metrics, which included TSS, PCC, Kappa, sensitivity, and specificity, further indicate the high‐quality performance of the models in both the training and cross‐validation phases (Table 1, Figures 2 and S4–S7). Among the five selected models, MaxEnt utilized all provided variables during the replicate runs, whereas BRT opted for the fewest variables, selecting only two of the 14 provided for both species (Table 1, Figures 2 and S4–S7).

**TABLE 1:** Model fit metrics for each of the participating modeling methods and for the final ensemble model for estimation of habitat suitability of *Arctonyx collaris* and *A. albogularis*.

Species	Model	Dataset	AUC	ΔAUC	PCC	TSS	Kappa	Specificity	Sensitivity
*A. collaris*	BRT	Train	0.972	0.037	91.3	0.821	0.793	0.917	0.905
		CV	0.935		87.3	0.721	0.696	0.891	0.830
	GLM	Train	0.990	0.056	95.3	0.906	0.887	0.954	0.952
		CV	0.934		88.6	0.753	0.723	0.898	0.855
	MARS	Train	0.985	0.031	95.3	0.906	0.887	0.954	0.952
		CV	0.954		87.9	0.728	0.706	0.898	0.830
	MaxEnt	Train	0.916	0.013	86.6	0.754	0.692	0.852	0.902
		CV	0.903		83.9	0.638	0.613	0.868	0.770
	RF	Train	0.963	0.005	92.7	0.854	0.825	0.926	0.929
		CV	0.968		92.0	0.78	0.793	0.955	0.825
*A. albogularis*	BRT	Train	0.987	0.094	96.3	0.929	0.925	0.958	0.971
		CV	0.893		84.2	0.692	0.681	0.850	0.842
	GLM	Train	0.946	0.036	89.0	0.778	0.775	0.896	0.882
		CV	0.910		85.3	0.700	0.699	0.850	0.850
	MARS	Train	0.959	0.024	87.8	0.757	0.751	0.875	0.882
		CV	0.935		84.4	0.678	0.672	0.870	0.808
	MaxEnt	Train	0.964	0.067	92.6	0.856	0.849	0.915	0.941
		CV	0.897		85.5	0.702	0.700	0.885	0.817
	RF	Train	0.931	0.012	87.8	0.757	0.751	0.875	0.882
		CV	0.943		83.0	0.655	0.653	0.830	0.825

*Note*: A total of five model algorithms were used with threshold of <0.75 AUC score. The models were maximum entropy (MaxEnt), random forest (RF), boosted regression tree (BRT), generalized linear model (GLM), and multivariate adaptive regression splines (MARS).

Abbreviations: AUC, area under curve; PCC, proportion correctly classified; TSS, true skill statistic; ΔAUC, change in area under curve (Training – Cross Validation).

### 3.2 Variables performance in the SDM

The ensemble model for *A. collaris* indicated that, on average (*μ*) across the five models, the primary contributor was the habitat variable evergreen broad leaf closed forest (euc_112), making up 62.76% of the model's influence, while annual mean temperature (bio_1) was the most significant contributing bioclimatic variable, accounting for 13.62% (Table 2). Among the topographic variables, slope (slp) made the highest contribution at 0.61%, and the anthropogenic variable Builtup/Urban (built‐up) contributed 3.65% to the model (Table 2). The least influential variable was normalized difference vegetation index (NDVI), with a contribution of 0.14%. Furthermore, the ensemble model for *A. albogularis*, revealed a different habitat variable, evergreen needle leaf closed forest (euc_111), as the highest contributor, which accounted for 57.02% of the contribution, while the highest contributing bioclimatic variable was temperature seasonality (bio_4), contributing 4.47%. Notably, temperature seasonality (bio_4) replaced the previously chosen mean diurnal range (bio_2) variable in *A. collaris* following the removal of multicollinearity among variables. Among the topographic variables, slope (slp) once again emerged as the highest contributor (7.89%), while the anthropogenic variable built‐up/urban (built‐up) made the lowest contribution to the model at 0.36% (Table 2).

**TABLE 2:** The mean percentage contribution of the covariates generated from the final ensemble model for two mainland hog badger species.

Species	Predictors	Predictors abbreviations	BRT	GLM	MARS	MaxEnt	RF	*μ* (mean)	*μ* (mean) %
*Arctonyx collaris*	Aspect	aspect	0.00000	0.00000	0.00000	0.00000	0.00456	0.00091	0.22
	Annual mean temperature	bio_1	0.07868	0.04232	0.02536	0.12612	0.01358	0.05721	13.62
	Precipitation seasonality (Coefficient of variation)	bio_15	0.00000	0.00000	0.00358	0.00024	0.00004	0.00077	0.18
	Precipitation of coldest quarter	bio_19	0.00000	0.01986	0.00000	0.02394	0.02734	0.01423	3.39
	Mean diurnal range (Mean of monthly (max temp − min temp))	bio_2	0.00000	0.00000	0.00450	0.00628	0.01822	0.00580	1.38
	Elevation	elevation	0.00000	0.00000	0.00000	0.01236	0.00000	0.00247	0.54
	Euclidean distance to evergreen needleleaf closed forest	euc_111	0.00000	0.00000	0.01928	0.01504	0.01368	0.00960	2.29
	Euclidean distance to evergreen broadleaf closed forest	euc_112	0.28272	0.43212	0.29856	0.24918	0.05460	0.26344	62.76
	Euclidean distance to deciduous broadleaf closed forest	euc_114	0.00000	0.08536	0.02540	0.02958	0.00452	0.02897	6.90
	Euclidean distance to shrubs	euc_20	0.00000	0.00000	0.00690	0.01162	0.02270	0.00824	2.00
	Euclidean distance to herbaceous wetlands	euc_90	0.00000	0.04216	0.00000	0.00900	0.00000	0.01023	2.44
	Euclidean distance to builtup/urban	euc_built	0.00000	0.04436	0.00576	0.00832	0.01824	0.01534	3.65
	normalized difference vegetation index	ndvi	0.00000	0.00000	0.00000	0.00135	0.00185	0.00060	0.14
	Slope	slp	0.00000	0.00560	0.00000	0.00714	0.00000	0.00255	0.61
*A. albogularis*	Aspect	aspect	0.00000	0.00000	0.05230	0.00022	0.00000	0.01050	2.25
	Annual mean temperature	bio_1	0.00000	0.00000	0.00000	0.00000	0.07494	0.01499	3.20
	Precipitation seasonality (Coefficient of variation)	bio_15	0.00000	0.00000	0.00000	0.00000	0.04164	0.00833	1.78
	Precipitation of coldest quarter	bio_19	0.00000	0.00000	0.00000	0.00000	0.01668	0.00334	0.71
	Temperature seasonality (standard deviation ×100)	bio_4	0.00000	0.00000	0.00000	0.00450	0.09998	0.02090	4.47
	Elevation	elevation	0.00000	0.00000	0.00000	0.00524	0.03328	0.00770	1.65
	Euclidean distance to evergreen needleleaf closed forest	euc_111	0.26960	0.39008	0.42722	0.23000	0.01662	0.26670	57.02
	Euclidean distance to evergreen broadleaf closed forest	euc_112	0.06010	0.00000	0.00000	0.01062	0.03338	0.02082	4.45
	Euclidean distance to deciduous broadleaf closed forest	euc_114	0.00000	0.00000	0.00000	0.00000	0.13328	0.02666	5.70
	Euclidean distance to shrubs	euc_20	0.00000	0.04768	0.00000	0.03414	0.00003	0.01637	3.50
	Euclidean distance to herbaceous wetlands	euc_90	0.00000	0.00000	0.00000	0.00524	0.11660	0.02437	5.21
	Euclidean distance to builtup/urban	euc_built	0.00000	0.00000	0.00000	0.00000	0.00832	0.00166	0.36
	Normalized difference vegetation index	ndvi	0.00000	0.02574	0.00000	0.00016	0.01668	0.00852	1.82
	Slope	Slp	0.00000	0.00000	0.00000	0.03452	0.14996	0.03690	7.89

### 3.3 Suitable habitat extent

**FIGURE 3:**
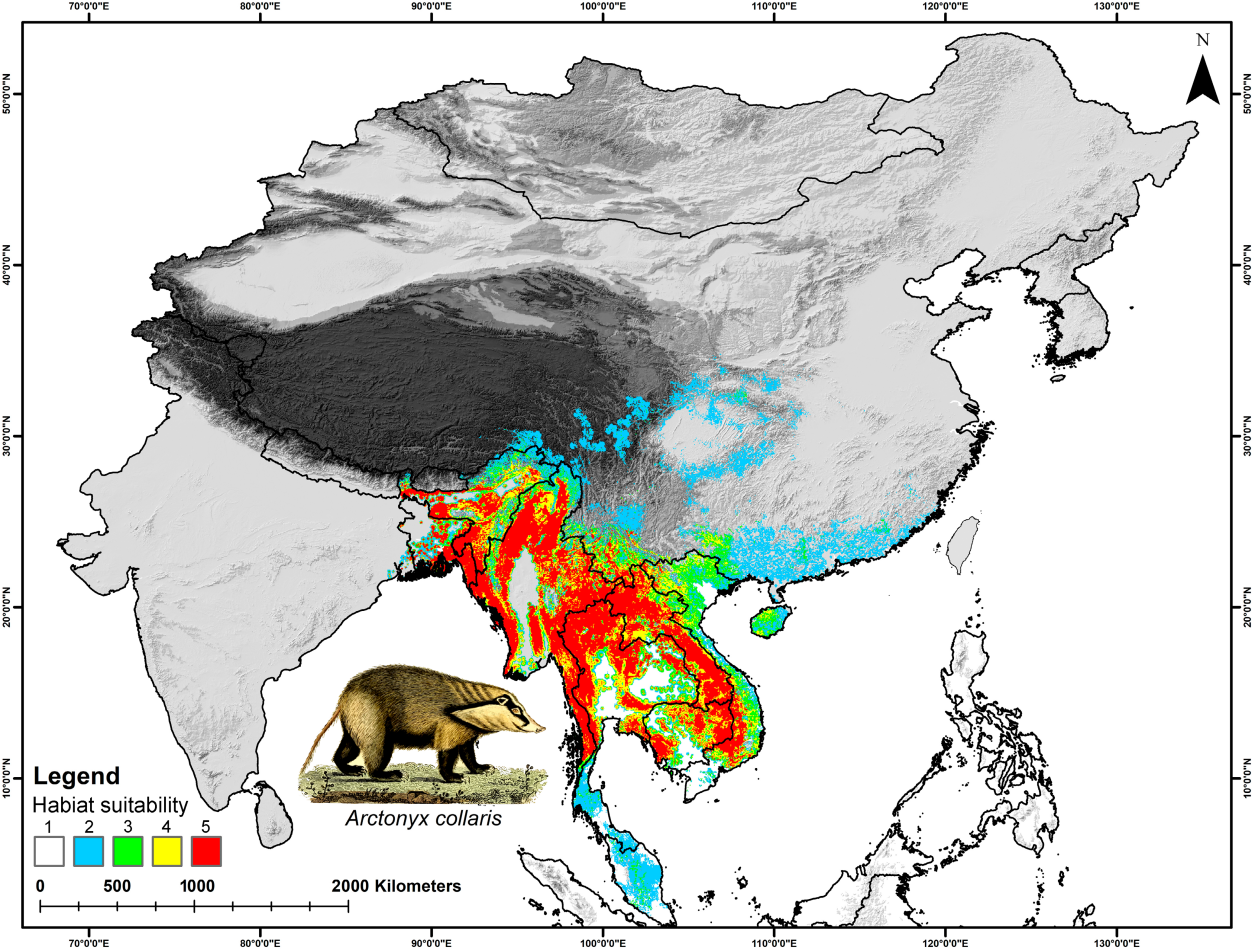
This figure shows the present suitable habitats for *Arctonyx collaris* in the study area. The five classes (1–5) defined in the map show the five model arguments used in the present study. The artwork of *A. collaris* reproduced from the free online media repository FreeSVG.org.

**FIGURE 4:**
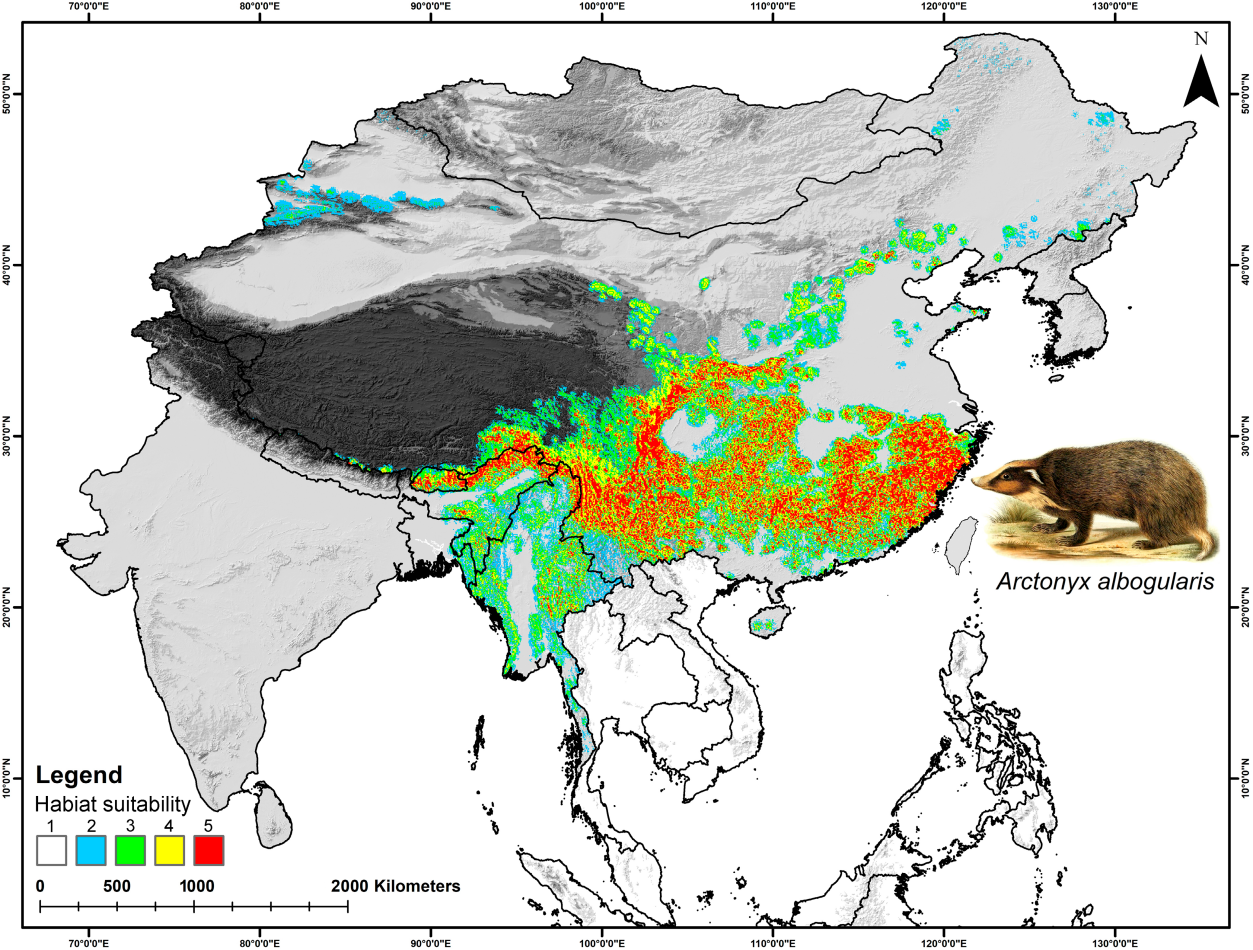
This figure shows the present suitable habitats for *Arctonyx albogularis* in the study area. The five classes (1–5) defined in the map show the five model arguments used in the present study. The artwork of *A. albogularis* reproduced from the free online media repository Wikimedia Commons.

**FIGURE 5:**
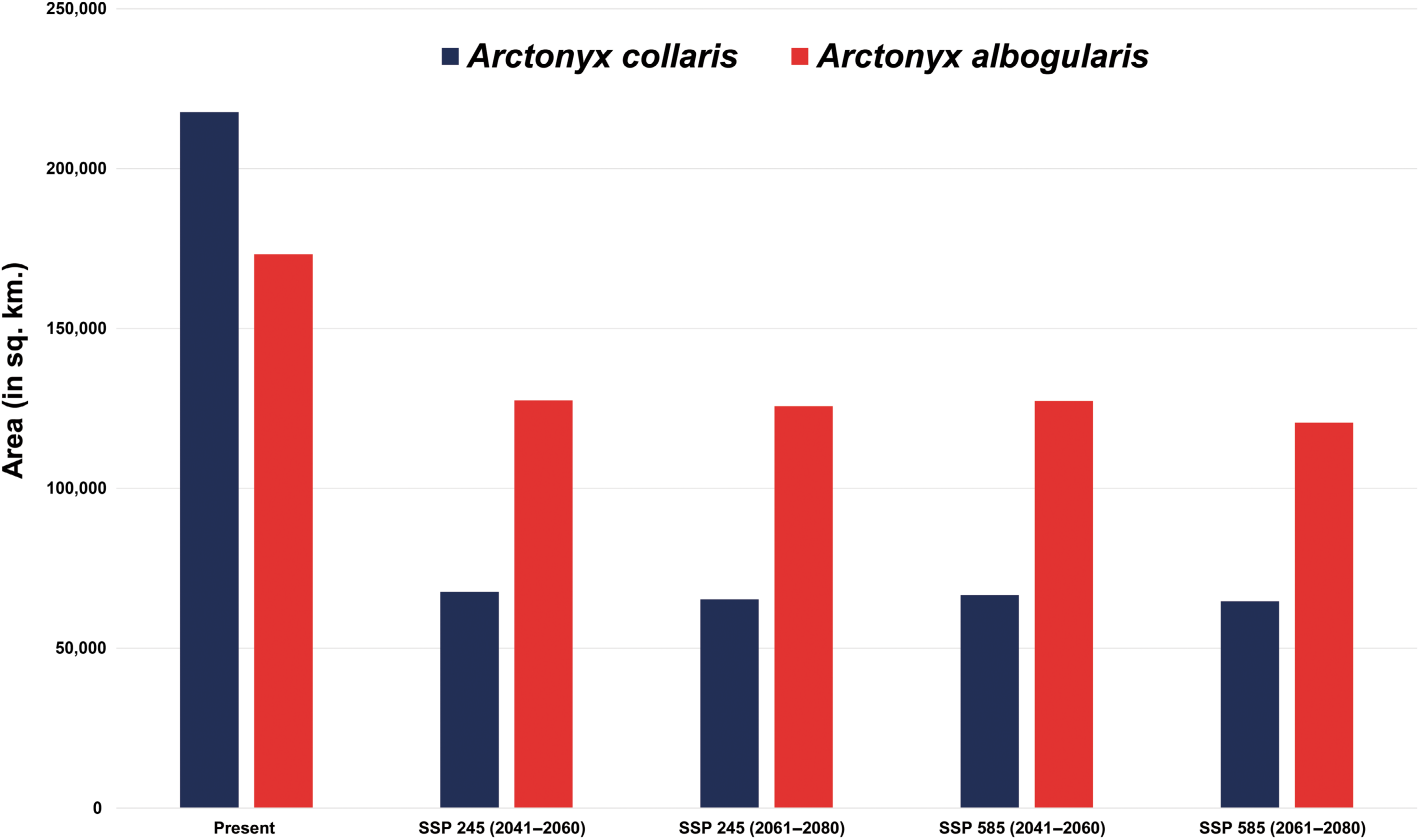
Area (km^2^) trend for *Arctonyx collaris* and *A. albogularis* in present and future scenarios.

**FIGURE 6:**
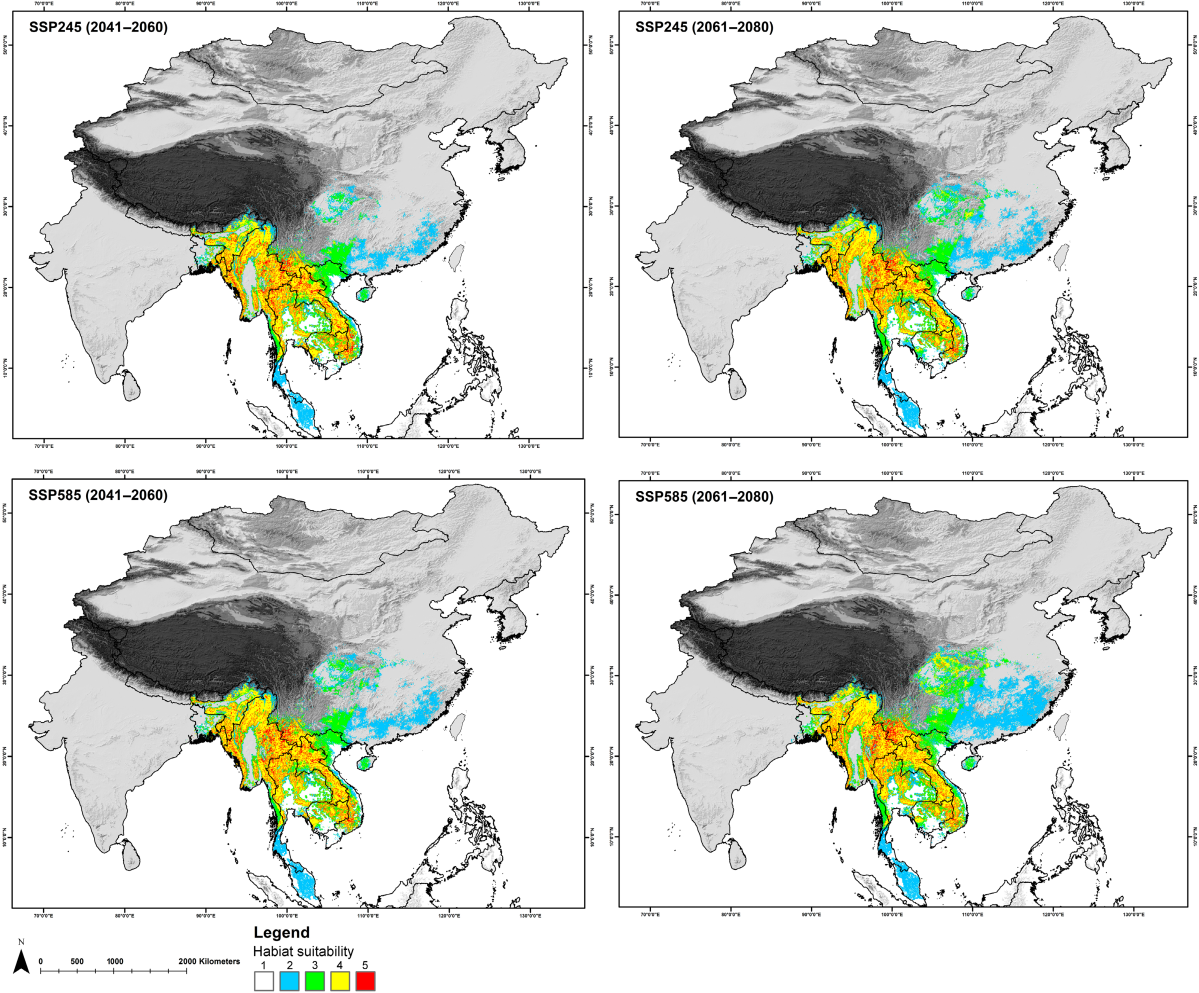
The figure shows the change in habitat suitability in future scenarios with the five model agreements (1–5) for *Arctonyx collaris*.

**FIGURE 7:**
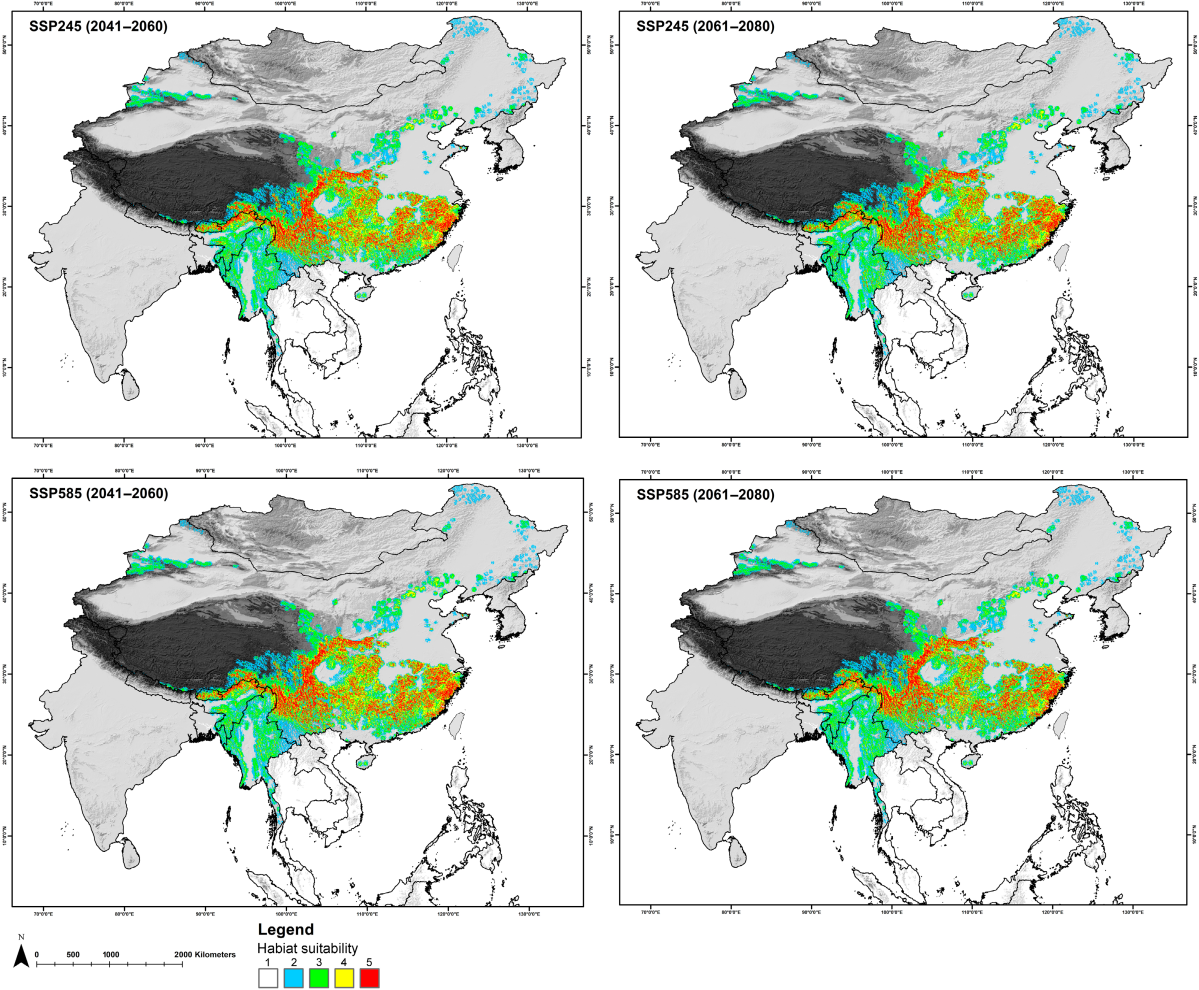
The figure shows the change in habitat suitability in future scenarios with the five model agreements (1–5) for *Arctonyx albogularis*.

In *A. collaris*, the total extent recognized by the IUCN Red List, which is about 412,627 km^2^ for *A. collaris*, the model identified only 217,728 km^2^ (52.76%) as presently suitable. While in *A. albogularis*, out of the total IUCN Red List extent of 1,014,758 km^2^, only 17.07% (173,241 km^2^) was deemed suitable (Figures 3, 4, 5; Table S1). The future climate change projections presented alarming outcomes for suitable areas across all scenarios, with a projected reduction from 68% to 70% for *A. collaris* and a decline of 26% to 30% for *A. albogularis* (Figures 6 and 7). Specifically, under the SSP245 scenario, habitat declines during the periods of 2041–2060 and 2061–2080 were estimated at 68.94% and 70.02%, respectively, for *A. collaris* (Figure 6). Similarly, the habitat declines for *A. albogularis* ranged between 26.39% and 27.40% (Figure 7). Furthermore, the high emission scenario SSP585 indicated even more severe habitat reductions, with declines of 69.43% and 70.32% during the same time intervals compared to the present scenario for *A. collaris*. Moreover, the reduction of suitable areas for *A. albogularis* during the same time was 26.49% and 30.43%, respectively.

### 3.4 Country level habitat dynamics

The model identified eight countries (India, Cambodia, Thailand, Vietnam, Laos, Bangladesh, Myanmar, China) within the distribution range of *A. collaris* as harboring suitable areas, while three countries (India, China, Myanmar) were suitable for *A. albogularis* (Table 3). Among them, Laos exhibited the highest average habitat suitability score (0.652) for *A. collaris*, whereas China showed the lowest score (0.002). Alternately, China displayed the highest habitat suitability score of 0.651 for *A. albogularis*, while Myanmar exhibited the lowest score of 0.031. This underscores the significant variability in habitat suitability across geographical regions for both species. Future projections in all scenarios indicated a decline of more than 70% in habitat suitability in Laos, with all countries experiencing a decrease for *A. collaris* (Table 3). However, China being an exception with exhibited a modest increase ranging from 4% to 14.22% across all future scenarios, with the highest percentage observed during the 2061–2080 period under the SSP585 scenario. Further in *A. albogularis*, all countries within its range experienced a notable decline in future climatic scenarios. Notably, the most predominant range country China witnessed a substantial decline of 90%–92%, respectively. Besides China, both extant ranges in India and Myanmar displayed a severe decline of over 30% in all future climate change scenarios for *A. albogularis* (Table 3).

**TABLE 3:** The table represents the countries with mean habitat suitability for *Arctonyx collaris* and *A. albogularis* in descending order for present and future climate change scenarios along with the percentage change in each future climate scenarios from the present.

Species	Country	Present	SSP 245 (2041–2060)	% change from present	SSP 245 (2061–2080)	% change from present	SSP 585 (2041–2060)	% change from present	SSP 585 (2061–2080)	% change from present
*A. collaris*	Laos	0.652	0.188	−71.25	0.180	−72.46	0.180	−72.35	0.151	−76.89
	Myanmar	0.534	0.156	−70.72	0.157	−70.68	0.157	−70.63	0.152	−71.45
	Thailand	0.401	0.110	−72.69	0.092	−77.19	0.102	−74.64	0.089	−77.77
	Cambodia	0.380	0.123	−67.68	0.099	−73.88	0.115	−69.63	0.105	−72.41
	Bangladesh	0.375	0.064	−82.95	0.064	−82.99	0.063	−83.11	0.063	−83.27
	India	0.253	0.071	−72.05	0.072	−71.68	0.069	−72.76	0.055	−78.12
	Vietnam	0.141	0.077	−45.47	0.077	−45.14	0.074	−47.68	0.077	−45.74
	China	0.002	0.003	+4.50	0.003	+5.92	0.003	+5.17	0.003	+14.22
*A. albogularis*	China	0.651	0.059	−90.89	0.049	−92.53	0.049	−92.43	0.047	−92.80
	India	0.111	0.070	−37.34	0.062	−44.37	0.067	−39.82	0.058	−47.85
	Myanmar	0.031	0.009	−70.72	0.012	−61.28	0.010	−68.00	0.008	−73.93

*Note*: “−” denotes decrease, whereas the increase is denoted by “+.”

### 3.5 Habitat quality and niche‐breadth and overlap

Given the severe reduction in suitable habitats for both species, significant changes have occurred in the geometry of these areas. Fragmentation is evident in both species, although *A. collaris* has been more severely affected than *A. albogularis* due to the substantial reduction in suitable patches (Table 4). Habitat assessment metrics revealed a high level of fragmentation but displayed contrasting results between the two species. For *A. collaris*, the NP has increased dramatically, showing an increase between 230% and 300%. This increase is attributed to the severe reduction in suitable habitat areas, resulting in small patch sizes, as reflected by a decrease of over 98% in the LPI in the future (Table 4). Consequently, edge metrics such as ED, TE, and LSI have increased, indicating an increase in edge and complex shape geometry. These patches are also relatively distant from each other, as indicated by the AI, which suggests that the proximity between suitable patches will decrease by over 60% in the future (Table 4). Overall, the decline in suitable areas has led to an increase in small, fragmented patches that are distant from each other due to fragmentation. However, interestingly, *A. albogularis* experienced a much lower degree of fragmentation compared to *A. collaris*. This is attributed to the less severe decline in suitable areas in contrast to *A. collaris*. Therefore, a decrease in NP of over 10% is observed for *A. albogularis*. Although these patches have reduced in size, they are still relatively large, as evidenced by a decrease of between 13% and 23% in LPI. Consequently, edge metrics (TE, ED, and LSI) have decreased, indicating less edge sharing between patches. As a result, the patches are in closer proximity compared to those of *A. collaris*, with AI declining only around 6% for *A. albogularis*. Overall, due to the relatively smaller decline in suitable areas, some patches have decreased in number as well as size, but they remain in closer proximity (Table 4).

**TABLE 4:** Habitat quality assessment of *Arctonyx collaris* and *A. albogularis* in present and future scenarios.

Species	Scenarios	NP	PD	LPI	TE	ED	LSI	AI
*A. collaris*	Present	1033	577,062.1	3.889	1369.662	7651.307	36.8958	83.9629
	SSP 245 (2041–2060)	3440	1,922,254	0.0483	1705.956	9532.794	81.532	34.8994
	SSP 245 (2061–2080)	3534	1,974,781	0.0565	1684.452	9412.63	81.7602	33.7369
	SSP 585 (2041–2060)	3635	2,031,219	0.0763	1721.286	9618.457	82.5622	33.5069
	SSP 585 (2061–2080)	4139	2,312,852	0.0209	1852.452	10,351.41	87.0748	31.4123
*A. albogularis*	Present	2607	1,456,342	0.8169	2843.61	15,885.19	82.2324	60.4342
	SSP 245 (2041–2060)	2186	1,221,160	0.6993	2264.808	12,651.84	76.1352	57.4043
	SSP 245 (2061–2080)	2243	1,253,001	0.6881	2234.442	12,482.21	76.1886	56.6772
	SSP 585 (2041–2060)	2186	1,221,160	0.7021	2258.34	12,615.71	76.1328	57.2093
	SSP 585 (2061–2080)	2273	1,269,760	0.6228	2148.888	12,004.28	74.9942	56.3404

Abbreviations: AI, aggregation index; ED, edge density; LPI, largest patch index; LSI, landscape shape index; NP, no. of patches; PD, patch density; TE, total edge.

While the current overlap of suitable areas remains minimal, encompassing approximately 1200 sq. km. However, this overlap is projected to decrease severely in the future, to around 162 sq. km. under the SSP585 (2061–2080) scenario Furthermore, the results of niche analysis revealed contrasting findings (Table 5). The Schoener's *D* niche overlap analysis conducted for both species yielded a value of 0.445, indicating an increase from the present to the future by 6%–27%, respectively. Additionally, the niche breadth was found to be 0.26 for *A. collaris* and 0.358 for *A. albogularis* at present. Interestingly, the niche breadth for both species exhibited an increasing trend (Table 5). For *A. collaris*, the increase ranged between 11% and 20%, respectively. However, the increase in niche breadth for *A. albogularis* was relatively modest, ranging between 0.3% and 0.8%, respectively.

**TABLE 5:** Assessment of niche breadth and niche overlap of *Arctonyx collaris* and *A. albogularis* in present and future scenarios.

Scenarios	Niche breadth		Niche overlap between two species
	*A. collaris*	*A. albogularis*	
Present	0.2600	0.3588	0.445766153
SSP 245 (2041–2060)	0.2910	0.3602	0.509520577
SSP 245 (2061–2080)	0.3011	0.3608	0.534903963
SSP 585 (2041–2060)	0.2991	0.3606	0.528109781
SSP 585 (2061–2080)	0.3131	0.3617	0.564738165

## 4 DISCUSSION

Small mammalian fauna plays a crucial role in maintaining ecological health and serve as indicators of ecological integrity in terrestrial landscapes (Nagy‐Reis et al., 2020). However, recent research has highlighted concerning trends in the loss of mammalian species and populations globally (Ceballos et al., 2005). This decline in mammalian diversity can largely be attributed to changes in land cover and environmental crises driven by increasing resource demands, particularly in biodiversity hotspots of Southeast Asia (Newbold et al., 2015). Furthermore, climate change has prompted small mammal species to respond by either dispersing to new, suitable habitats, adapting to changing conditions, or facing extinction (Quintero & Wiens, 2013). In addition to these challenges, certain genera, including *Arctonyx*, have not been thoroughly assessed regarding the adverse impacts of climate change (Kalle et al., 2013; Proulx et al., 2016). Hence, the outcomes of this study represent the most comprehensive analysis to date of the effects of climate change on the two mainland hog badger species.

Despite the extensive geographic range occupied by both mainland hog badger species across Asia, the present study revealed that only 217,728 km^2^ (52.76%) of this extent as suitable for *A. collaris*, out of the total range (412,627 km^2^) designated by the IUCN Red List. Similarly, the analysis found that only 17.07% (173,241 km^2^) of the IUCN extent was suitable for *A. albogularis* (Figures 3, 4, 5, 6, 7; Table S1; Helgen & Chan, 2016; Zhang, 1997). The current assessment also reveals a troubling prospect, with a projected decline of 26%–70% in suitable areas for both mainland hog badgers in the forthcoming decades. This concerning trend can be attributed to climatic changes and their impacts on these species. The results highlight bioclimatic variables such as annual mean temperature, temperature seasonality, and precipitation in the coldest quarter as some of the highest contributing factors. These results align with previous studies that have identified temperature predictors as significant contributors to their decline, underscoring the critical need for targeted conservation strategies to mitigate these impacts (Dutta et al., 2022; Morueta‐Holme et al., 2010).

The anticipated declines in the current study are also corroborated by previous studies of other mustelid species around the world, which have shown comparable reductions (Barlow et al., 2021). For instance, a study focused on the Marbled Polecat, *Vormela peregusna* in China revealed that climate change will profoundly impact the species (Cheng et al., 2023). The projections indicated a substantial reduction in suitable habitats, where currently suitable areas become unsuitable in the future. Similarly, research on the Yellow‐throated Marten, *Martes flavigula* highlighted the severe impact of climate change and habitat loss in the Himalayan region (Dutta et al., 2022). The projections suggested a decline of over 50% in its range, particularly in the eastern Himalayas. Additionally, the distribution of *M. flavigula* may abandon current habitats and seek new refuges. Similar studies conducted in South America and Europe also forecast significant future declines in mustelid species (Lamelas‐López et al., 2020; Schiaffini, 2022). These findings align with the current study findings, indicating substantial losses for the mainland hog badger in their particular habitat. Such habitat decline poses an extremely serious challenge to the conservation of these species, and hence it is strongly recommended that measures be taken, such as long‐term monitoring for the conservation of vulnerable animals. Additionally, it is vital to protect the primary habitats determined by the model in this study: the Evergreen Broad Leaf Closed Forest and the Evergreen Needle Leaf Closed Forest (Table 2). This is crucial for conserving both mainland badgers in the biodiversity hotspots in Southeast Asia, which faces significant forest vegetation losses (>6 million hectares), thus aligning with the recommendation of the IUCN assessment (Hu et al., 2021). This looming threat of irreversible biodiversity decline is particularly pronounced in these landscapes, which are already heavily fragmented due to ongoing habitat loss that reduces patch sizes and increases distances between them, disrupting connectivity and exacerbating edge effects (Taubert et al., 2018). Therefore, increased habitat fragmentation is associated with higher rates of species decline due to additional habitat loss. The present study aligns with these findings, as the extensive reduction in suitable habitat has led to significant alterations in spatial configurations, resulting in noticeable fragmentation that adversely affects both species (Table 4). For *A. collaris*, this shift has resulted in a substantial rise in the NP, consequently diminishing patch sizes in the future. As a consequence, metrics gauging edge characteristics have displayed an increase, denoting more intricate edge configurations. Intriguingly, *A. albogularis* exhibited less fragmentation compared to *A. collaris*, with a reduction of over 10% in the number of patches observed. Despite this decline, the patches remained relatively large, resulting in a decrease of between 13% and 23% in the LPI (Table 4). Such habitat fragmentation induced by climate change is evident in various mustelids around the world. These fragmented areas are insufficiently protected and do not fall under legal jurisdiction (Almasieh & Cheraghi, 2022). Hence, beyond the natural suitable areas, this study advocates research in protected areas will yield a more comprehensive understanding of hog badgers, facilitating sustainable conservation efforts through both in situ and ex‐situ initiatives.

Moreover, previous studies have primarily emphasized hunting and wildlife trade as significant threats to the hog badgers, neglecting to address climate change and habitat fragmentation (Duckworth et al., 1999; Helgen et al., 2008). The IUCN assessment categorized *A. collaris* as “Vulnerable,” which overlooked critical aspects like climate change and habitat fragmentation (Wheatley et al., 2017). Regardless of the decreasing population trend, *A. albogularis* was categorized as “Least Concern” by the IUCN assessment, which is also threatened by hunting pressure (Helgen & Chan, 2016). Given that the current study identified comparable patterns of habitat decline and fragmentation in the future, alongside similar threats evaluated by the IUCN Red List for both species. It is therefore suggested that the IUCN may reassess the status of *A. albogularis*, akin to its counterpart, *A. collaris*, which is a threatened taxon. Hence, future IUCN assessments ought to integrate the impacts of climate change into their evaluation criteria, incorporating changes in species distribution resulting alongside population dynamics and human‐induced pressures on populations (Cheng et al., 2023). This is imperative, as the IUCN Red List serves as a primary standard in wildlife conservation and a reference for prioritizing conservation efforts and ecological research (Pimm et al., 2014; Tianpei et al., 2022). Hence, the present study will be helpful to the IUCN/SSC SMSG for a detailed evaluation of both these mainland hog badger species.

Nevertheless, the IUCN assessment found minimal geographical overlap between these species (Proulx et al., 2016), a finding corroborated by the present study. Despite the limited overlap, Schoener's *D* niche overlap analysis yielded a high value of 0.445, which is noteworthy. Additionally, the study predicts an increase in this overlap from the present to the future. This factor might also account for previous uncertainties surrounding the differentiation of the two species (Allen, 1938; Choudhury, 2013; Pocock, 1941). In addition to the heightened niche overlap observed between the two species, it was noted that the niche breadth expands in future climate change scenarios. This finding aligns with research conducted on various mustelids in North America and Southeast Asia (Kupferman et al., 2021; Sibarani et al., 2022). Given that competition among species within the same ecological niche can modulate populations and affect community composition, niche partitioning serves as a mechanism for sympatric species to coexist by utilizing space, time, and resources differently. It arises from interspecific competition among closely related species, enabling their coexistence. Therefore, it is plausible that they have broadened their niche breadth to alleviate resource competition between the two mainland hog badgers.

Given that earlier studies have extensively emphasized the necessity for targeted conservation efforts for this genus *Artctonyx* within its natural habitat, it has become increasingly important to enhance crucial management efforts for hog badgers, especially in countries where hunting and illegal trade intersect with other detrimental factors impacting their populations (Kurek et al., 2022). Hence, considering the distinct legal frameworks of each country, it is crucial to formulate an effective conservation strategy by identifying suitable habitat for focused efforts. Specifically, legal forest authorities in Cambodia, Myanmar, and Vietnam should enlist the badger species in their wildlife protection laws to ensure the protection of hog badgers and other small mammals. Additionally, the China Red List should differentiate between the two hog badger species, as they are currently listed as a monotypic genus, to ensure species‐specific protection. Furthermore, countries such as Bhutan, China, Myanmar, and Vietnam should conduct field studies to confirm the presence of these two species in areas marked as “possibly extinct” or “presence uncertain.” The present study identifies areas of suitable habitat loss due to climate change as well as new regions that may become suitable due to climatic shifts. This finding is crucial, as regions lost to climate change require stringent protection, particularly to safeguard surrounding forested areas by bringing them under legal law of protection. Simultaneously, newly suitable areas must prioritize the preservation of their forest lands to ensure future viability, potentially serving as refuges or translocation sites for these small mammals. Therefore, strategic planning at the landscape level is essential, potentially incorporating transboundary cooperation. Moreover, climate change has resulted in the emergence of numerous small, viable habitat patches within the anticipated future distribution range of these species, causing fragmentation of the currently larger habitat areas. This fragmentation is evident throughout their entire distribution range but is especially pronounced in the northeastern states of India, including Assam, Tripura, and Manipur, as well as in neighboring countries such as Myanmar (northern part), Thailand, and Vietnam. However, unlike northern Myanmar, the southern part of Myanmar and adjacent regions in China still retain some extensive habitat patches. These larger, contiguous areas could serve as crucial refuges for conservation efforts and should be prioritized in conservation planning. Therefore, maintaining ecological corridors between fragmented patches of suitable habitat is essential for preserving gene flow among different populations of this small mammal. These corridors would facilitate dispersal and movement across the landscape, thereby reducing the risk of inbreeding depression and promoting genetic diversity. This would enhance the resilience of these species to climate change, thus ensuring continuous monitoring and adaptive management in response to ongoing environmental changes and maintaining ecological balance. Additionally, international and national law enforcement agencies should monitor this region, particularly in northeast India (Assam, Manipur, Nagaland, etc.) to Myanmar and Malaysia, as these areas are regarded as significant wildlife trafficking routes. Moreover, it is also essential for international law enforcement agencies to ban and sanction the use of hog badgers for various human needs (bushmeat, hair brushes, etc.), thus reducing their illegal demand in international markets and declining hunting in South and Southeast Asia. These strategies require a comprehensive and multifaceted approach that actively involves local communities, conservationists, and governmental bodies. It is essential to foster awareness and understanding among the local people regarding the importance of conserving these small mammals. This educational effort must be directed toward individuals in deprived and remote areas, where awareness of the ecological roles of wildlife may be limited. Therefore, by elucidating the benefits these animals provide and clarifying that they pose no harm to humans, it is possible to mitigate misconceptions and reduce instances of retaliation. For instance, addressing the issue of retaliatory killings due to perceived threats to crops or mistaken identity with wild boars is crucial. In addition, establishing local conservation groups or response units is vital for implementing effective conservation measures that focus on preserving not only the targeted species but also the broader fauna within their habitats. This approach ensures that conservation efforts are well‐coordinated, integrate community engagement, and ultimately lead to more effective and lasting outcomes for both the species and their habitats. Moreover, the extent of two mainland hog badgers encompasses three major biodiversity hotspots (Eastern Himalaya, Southwest China, and Indo‐Burma) and numerous protected areas, that require the utmost legal protection and coordination among countries for integrated protection (Marchese, 2015; Myers et al., 2000). Lastly, in the context of mammalian research, there is a need for regular assessments of this small mammal to fill gaps in knowledge, such as presence information and population status, through an integrated approach encompassing morphology, ecology, and genetics. This will benefit both researchers and conservation agencies, prompting them to encourage and support young researchers in protecting this small mammal in the wild and preventing it from the brink of extinction.

## 5 CONCLUSION

In summary, this study highlights the pressing necessity for tailored conservation initiatives aimed at safeguarding the mainland hog badgers, *A. collaris* and *A. albogularis*, across mainland Asia. The research reveals that the suitable habitats for both species are increasingly imperiled by climate change, with substantial projected declines expected in the coming decades. While past attention has predominantly focused on hunting and wildlife trade as primary threats, this study emphasizes the crucial need to incorporate considerations of climate change and habitat fragmentation into future conservation assessments. The findings illustrate that these dwindling suitable habitats are experiencing significant fragmentation, further exacerbating the challenges faced by these species. Moreover, given the alarming habitat loss, this study advocates for a reevaluation of the IUCN status of *A. albogularis*. Specifically, it identifies several countries, including China, Myanmar, Laos, Vietnam, and India, where targeted conservation efforts are urgently needed for both mainland hog badger species. Such efforts would involve designating specific areas within their distribution range as conservation sites and designate ecological corridors between the viable fragmented patches for protecting the gene pool of these species. Furthermore, the study recommends that the IUCN Specialist Group integrate the impacts of climate change and habitat fragmentation into their species assessment methodologies. Ultimately, incorporating these insights into conservation strategies can empower authorities to strengthen protection measures for these species, thereby contributing to the preservation of biodiversity within their habitats.

## AUTHOR CONTRIBUTIONS

**Imon Abedin:** Data curation (equal); formal analysis (equal); methodology (equal); software (equal); writing – original draft (equal). **Tanoy Mukherjee:** Conceptualization (equal); formal analysis (equal); investigation (equal); resources (equal); software (equal); supervision (equal); writing – original draft (equal). **Ah Ran Kim:** Data curation (equal); investigation (equal); methodology (equal); project administration (equal). **Soo Rin Lee:** Data curation (equal); funding acquisition (equal); investigation (equal); project administration (equal); validation (equal). **Hyun‐Woo Kim:** Funding acquisition (equal); resources (equal); validation (equal); visualization (equal); writing – review and editing (equal). **Shantanu Kundu:** Conceptualization (equal); project administration (equal); resources (equal); supervision (equal); visualization (equal); writing – review and editing (equal).

## FUNDING INFORMATION

This research was supported by the Basic Science Research Program through the National Research Foundation of Korea (NRF) funded by the Ministry of Education (2021R1A6A1A03039211).

## CONFLICT OF INTEREST STATEMENT

The authors declare no competing interests.

## Supporting information


Data S1.

## Data Availability

Data used for the analysis were sourced from open‐access resources.
