# Supplementary material for: Fragile futures: Evaluating habitat and climate change response of hog badgers (Mustelidae: Arctonyx) in the conservation landscape of mainland Asia
Source: Ecol Evol. 2024 Aug 14;14(8):e70160. doi: 10.1002/ece3.70160 (PMC11322595; doi:10.1002/ece3.70160)
Supplement: Supplementary file 1 — Data S1. [file ECE3-14-e70160-s001.docx]

**SUPPORTING INFORMATION**


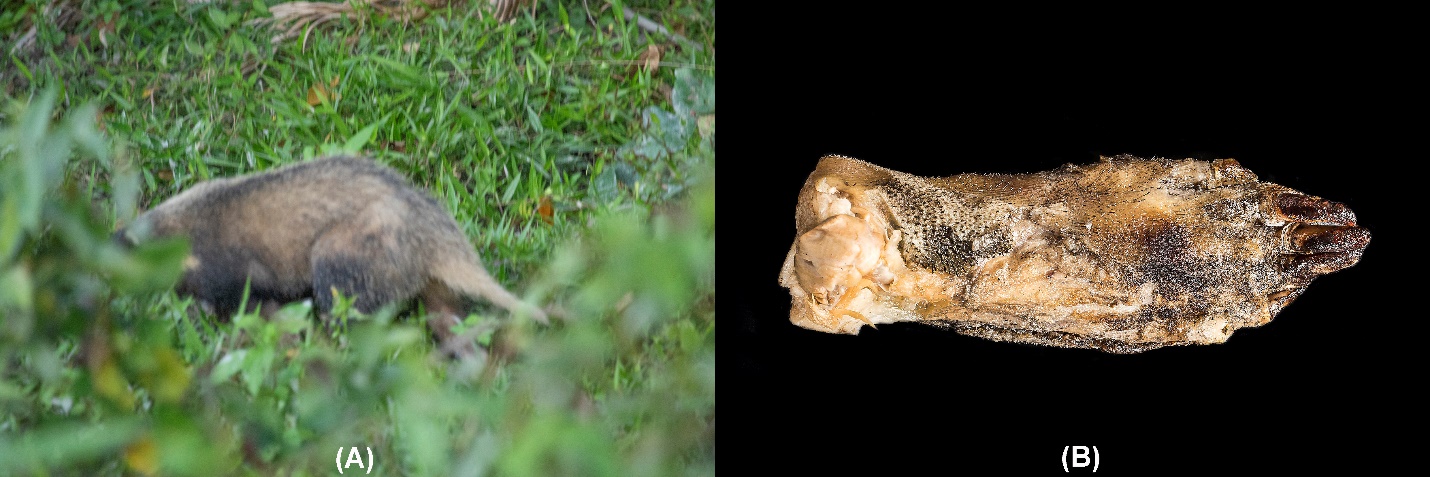


**Figure S1** (A) Photographic record of *A. collaris* documented in Kaziranga National Park, Assam, and (B) Body part of *A. collaris* found in Bunning Wildlife Sanctuary, Manipur, in Northeast India. Photo Credits: Imon Abedin and Shantanu Kundu.


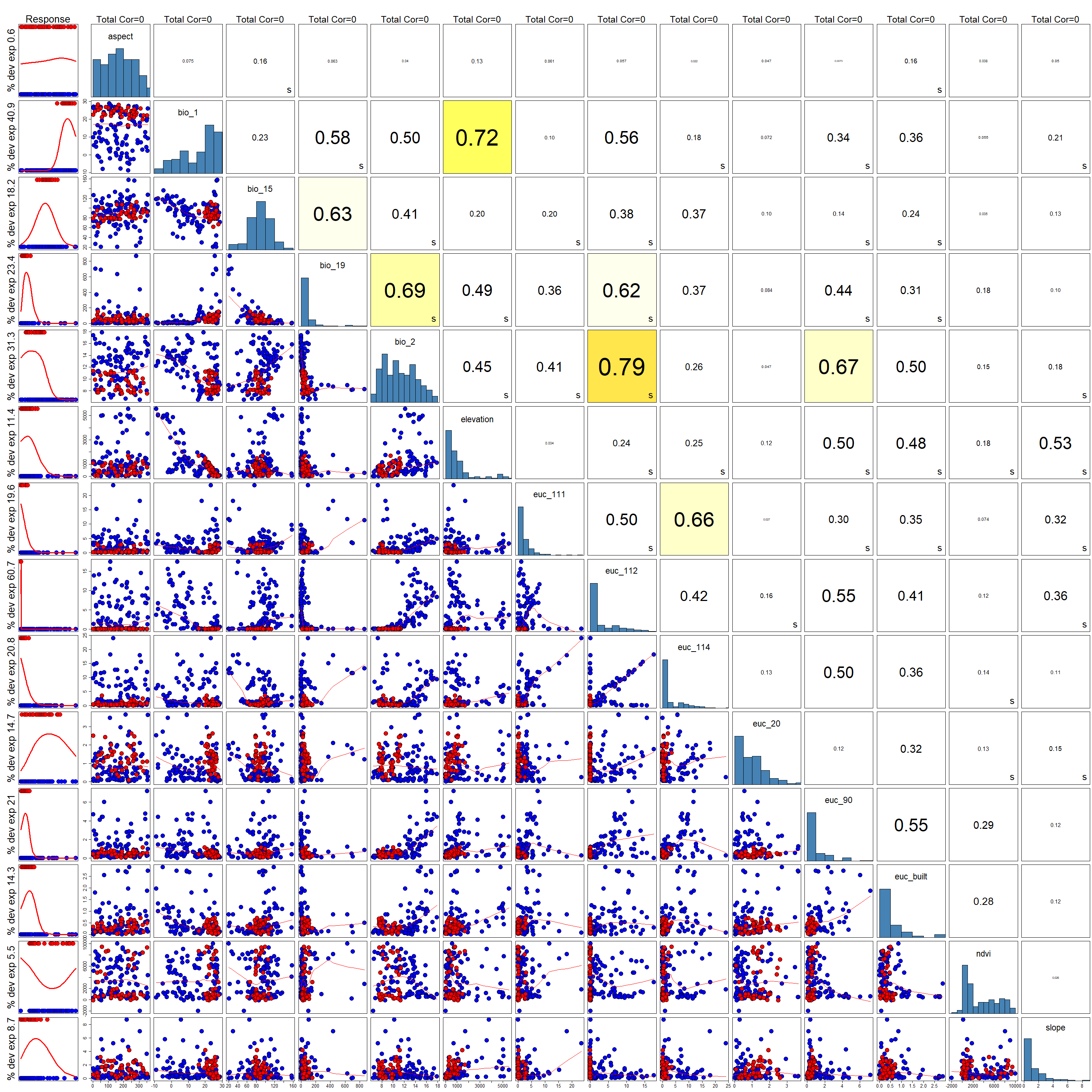


**Figure S2** Figure showing the correlation between the covariates chosen for final model for *A. collaris*.


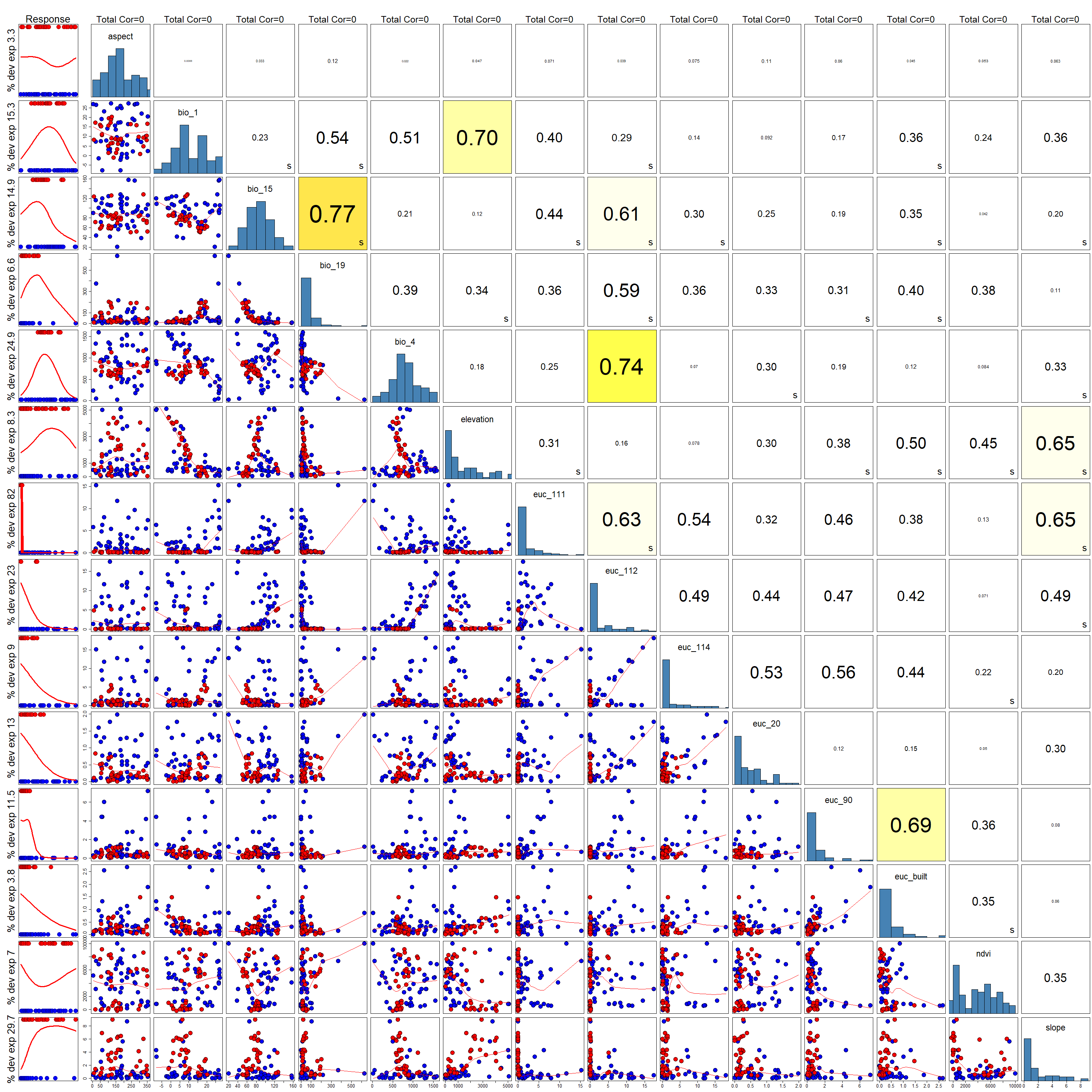
 **Figure S3** Figure showing the correlation between the covariates chosen for final model for *A. albogularis*.


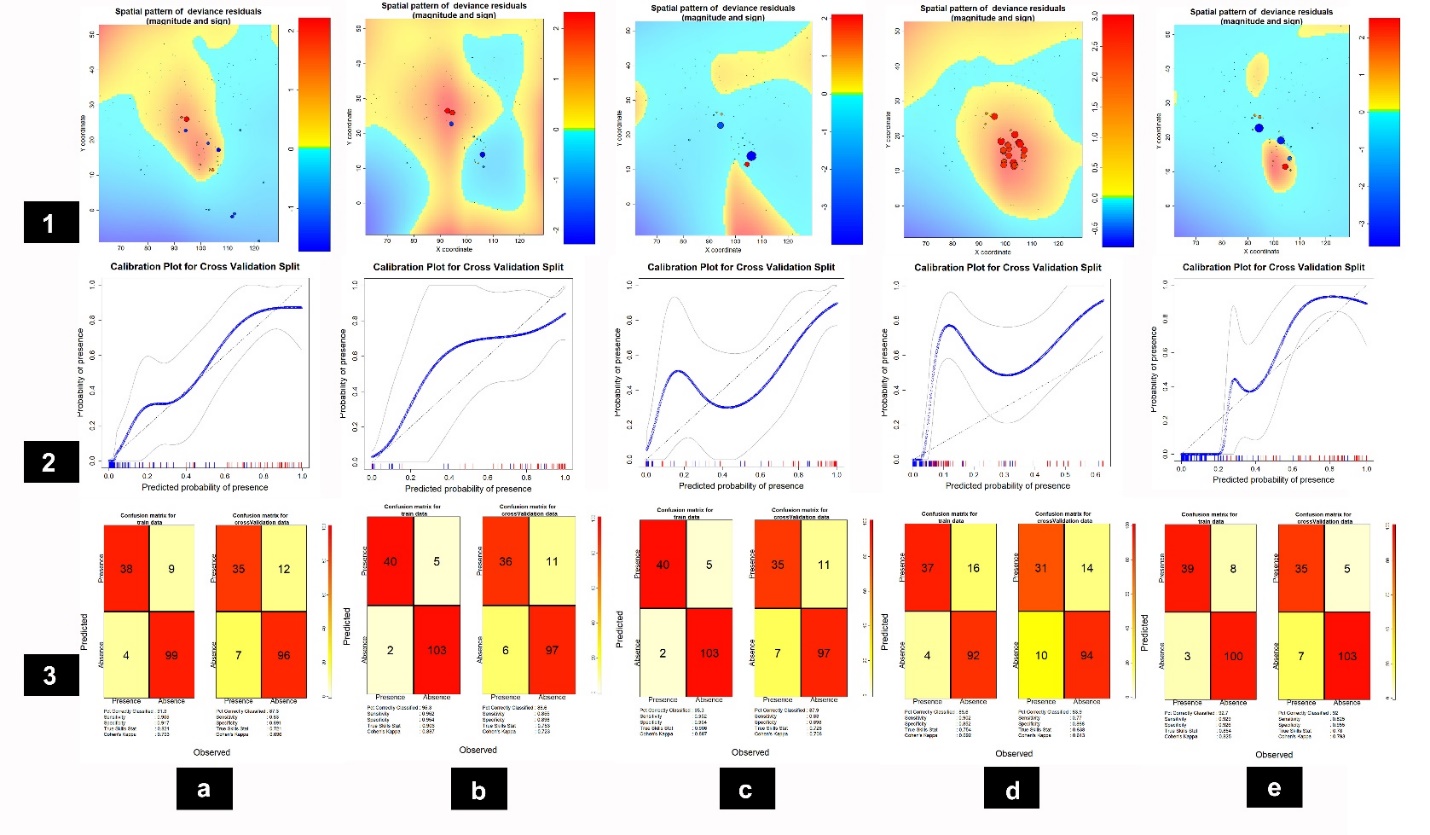


**Figure S4** Confusion matrixes, Model Calibration plots and Residual plots for *A. collaris*. Row 1 represents spatial pattern of residuals where colour ramp indicates the magnitude of deviance and size represents the quantity. Row 2 represents model calibration plot across all 5 different model for cross-validation split. Row 3 represents confusion matrix for all 5 models, plotted by observed vs. predicted where colour ramp from lowest value 0% (white) to 100% (red) indicates the quantification of particular pair types. Column a. represents plots for BRT, Column b. represents plots for GLM, Column c. represents plots for MARS, Column d. represents plots for MaxEnt and Column e. represents plots for RF.


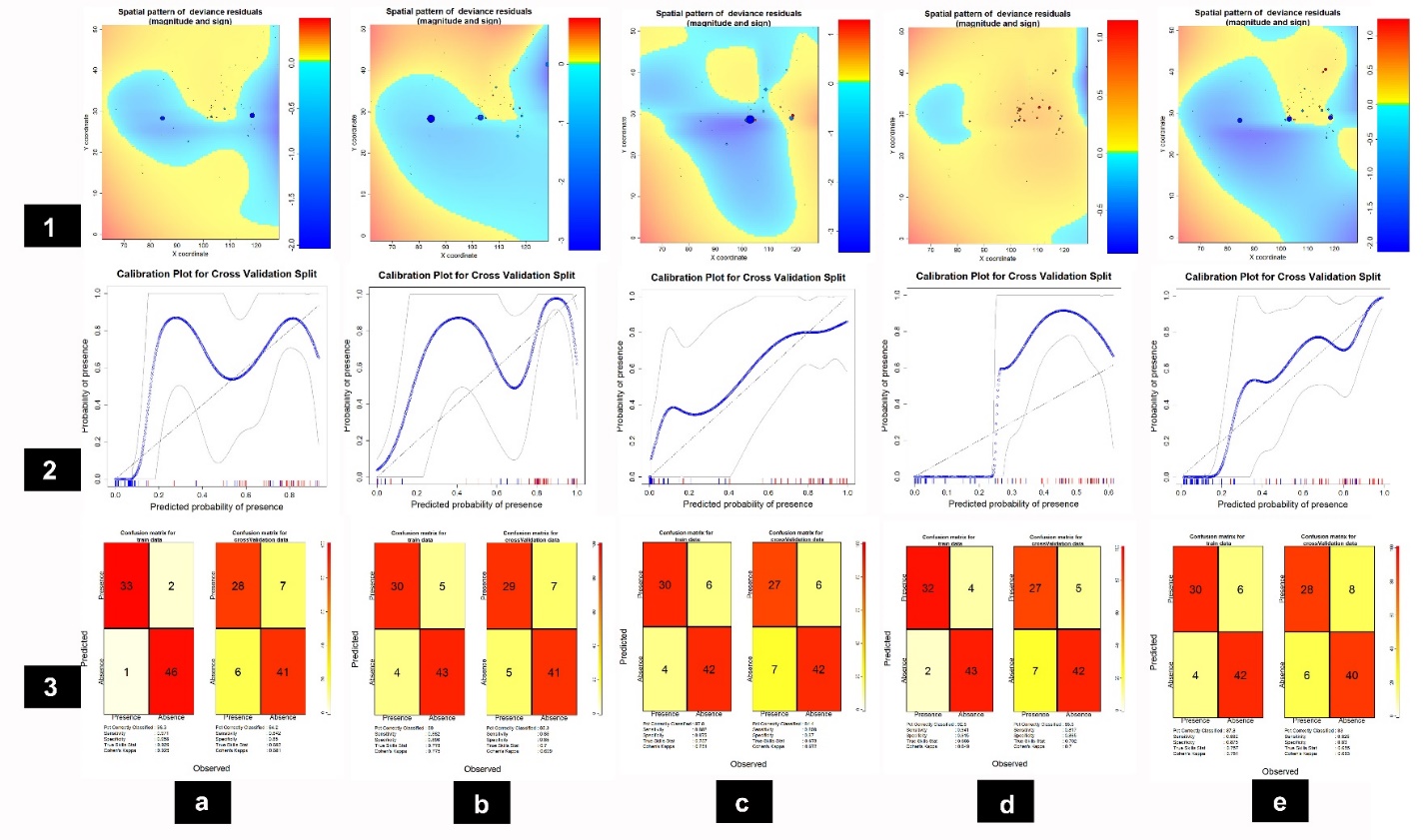


**Figure S5** Confusion matrixes, Model Calibration plots and Residual plots for *A. albogularis*. Row 1 represents spatial pattern of residuals where colour ramp indicates the magnitude of deviance and size represents the quantity. Row 2 represents model calibration plot across all 5 different model for cross-validation split. Row 3 represents confusion matrix for all 5 models, plotted by observed vs. predicted where colour ramp from lowest value 0% (white) to 100% (red) indicates the quantification of particular pair types. Column a. represents plots for BRT, Column b. represents plots for GLM, Column c. represents plots for MARS, Column d. represents plots for MaxEnt and Column e. represents plots for RF.


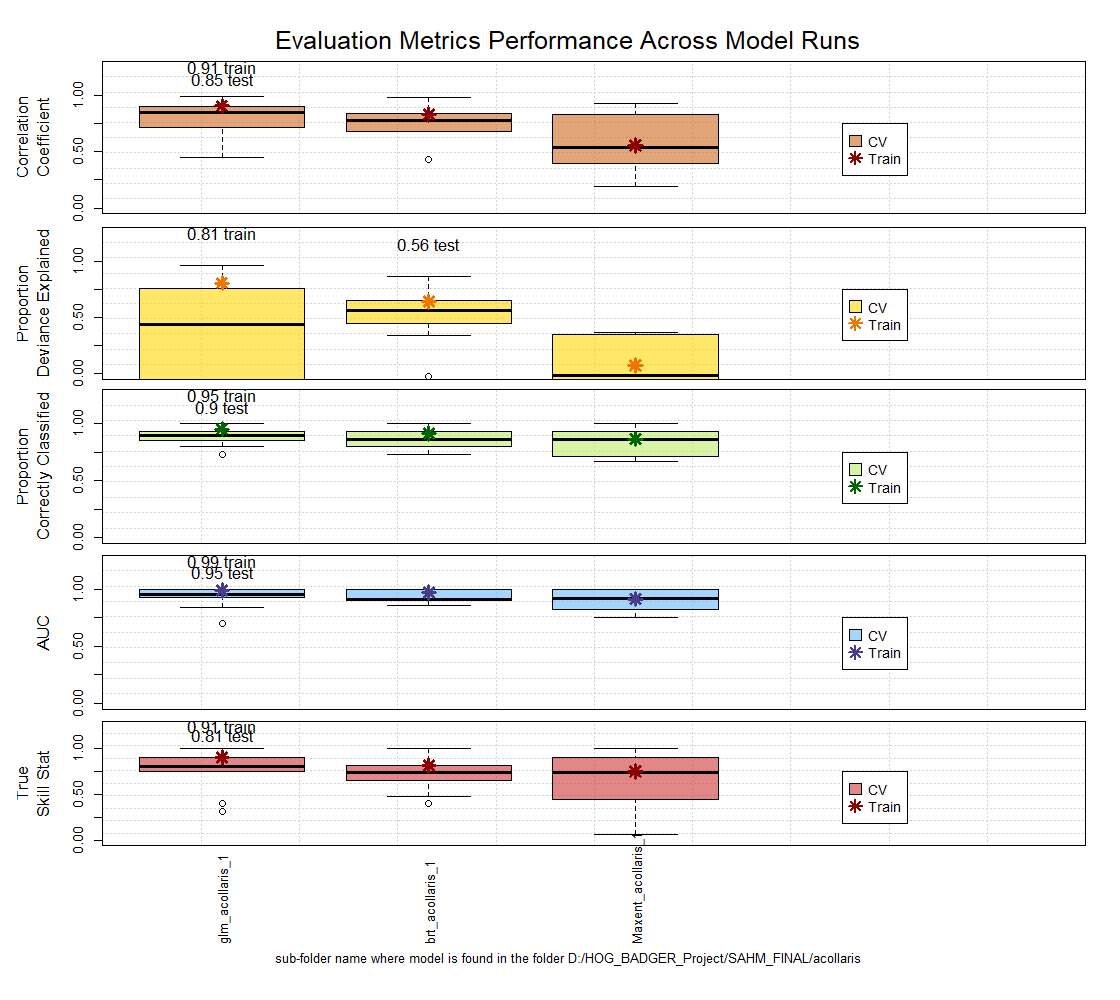
 **Figure S6** Evaluation Matrix performance across model runs for *A. collaris*. Brown - represents the correlation coefficient among the 5 different models. Yellow - represents the proportion of deviance explained; Green - represents the Proportion of correctly classified; Blue - represents Area under curve (AUC) and Pink - represents true skill statistics.

**
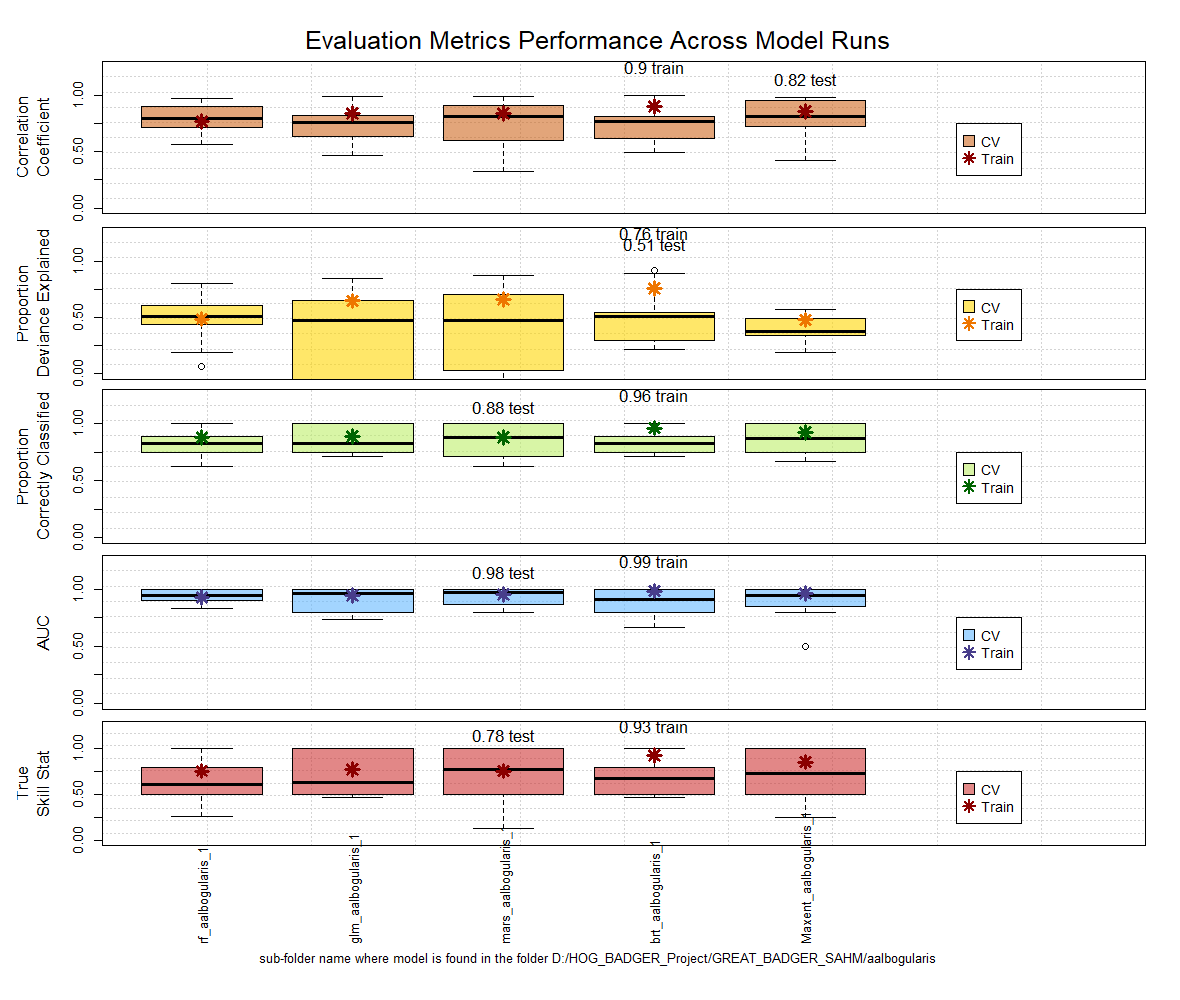
**

**Figure S7** Evaluation Matrix performance across model runs for *A. albogularis*. Brown - represents the correlation coefficient among the 5 different models. Yellow - represents the proportion of deviance explained; Green - represents the Proportion of correctly classified; Blue - represents Area under curve (AUC) and Pink - represents true skill statistics.

**Table S1.** Percentage of area change in Km^2^ for two mainland hog badgers. “-” denotes loss/reduction.

| **Species** | **Present (in sq. Km.)** | **SSP 245 (2041-2060) (in sq. Km.)** | **% change from Present** | **SSP 245**  **(2061-2080) (in sq. Km.)** | **% change from Present** | **SSP 585 (2041-2060) (in sq. Km.)** | **% change from Present** | **SSP 585 (2061-2080) (in sq. Km.)** | **% change from Present** |
| --- | --- | --- | --- | --- | --- | --- | --- | --- | --- |
| 1. *collaris* | 217728 | 67617 | -68.94 | 65272 | -70.02 | 66564 | -69.43 | 64625 | -70.32 |
| 1. *albogularis* | 173241 | 127516 | -26.39 | 125779 | -27.40 | 127341 | -26.49 | 120523 | -30.43 |
| Overlap of two Species | 1200 | 380 | -68.33 | 270 | -77.50 | 210 | -82.50 | 162 | -86.50 |
